# Monoterpenoids from the Roots of *Liquidambar formosana* (Formosan Sweet Gum) Exhibit Senomorphic Activity Against Cellular Senescence

**DOI:** 10.3390/nu17213321

**Published:** 2025-10-22

**Authors:** Minh Thi Tuyet Le, Quang Huy Vu, Van-Hieu Mai, Jorge Eduardo Ponce-Zea, Seri Choi, Jin-Pyo An, Won-Keun Oh

**Affiliations:** Research Institute of Pharmaceutical Sciences, College of Pharmacy, Seoul National University, Seoul 08826, Republic of Korea; lethituyetminh19289@gmail.com (M.T.T.L.); 2023-23730@snu.ac.kr (Q.H.V.); maihieu@snu.ac.kr (V.-H.M.); jepz210689@snu.ac.kr (J.E.P.-Z.); loyalmintt2@naver.com (S.C.); ntopjp77@gmail.com (J.-P.A.)

**Keywords:** *Liquidambar formosana*, cellular senescence, monoterpenoids, senomorphic effects, SASP inhibition

## Abstract

Background/objectives: Cellular senescence is a hallmark of aging that contributes to tissue dysfunction and age-related diseases. This process is characterized by the activation of the cyclin-dependent kinase inhibitor p16^INK4A^ and the secretion of pro-inflammatory factors collectively known as the senescence-associated secretory phenotype (SASP). In this study, we used human lung-derived cells, including A549 and IMR90 fibroblasts, to identify bioactive compounds from the roots of *Liquidambar formosana* that suppress p16^INK4A^ activity and attenuate SASP expression. Methods: Bioactivity-guided isolation was performed to obtain target compounds. The structures of the new compounds were elucidated using extensive 1D and 2D NMR spectroscopic analyses as well as high-resolution mass spectrometry. All isolated compounds were evaluated for their ability to inhibit p16^INK4A^, a key regulator of the cell cycle and an important tumor suppressor protein. Results: Two previously undescribed monoterpenoids (**1** and **2**), characterized as cinnamic acid esters with a monoterpene-derived core, were isolated from the roots of *L. formosana*, along with six known compounds (**3**–**8**). Notably, compound **3** exhibited promising inhibition of p16^INK4A^ with an IC_50_ value of 3.9 μM. Furthermore, this compound attenuated the senescence phenotype, as demonstrated by β-galactosidase staining and RT-qPCR analysis. This represents the first report identifying bioactive monoterpenoids from *L. formosana* that inhibit aging-related biomarkers such as p16^INK4A^. Conclusions: These results suggest that cinnamic acid-conjugated monoterpenoids may serve as interesting lead structures for the development of agents targeting the p16^INK4A^ pathway for the treatment of aging-associated diseases. Further studies will be required to clarify the mechanisms of action of this compound and to evaluate its in vivo efficacy.

## 1. Introduction

The genus *Liquidambar*, commonly known as sweet gum, belongs to the family Altingiaceae and comprises approximately 15 species of deciduous trees widely distributed across East Asia, North America, and the Mediterranean region. Among these, *Liquidambar styraciflua* (American sweet gum) is the most widely recognized species, primarily found in North America [1]. Another important species is *Liquidambar formosana* (Formosan sweet gum), which is native to East Asia and has been traditionally used in folk medicine [2]. Recent studies have revealed several biological activities associated with *L. formosana*. The balsam of this species exhibits antifungal activity, with compounds such as 3α,25-dihydroxyolean-12-en-28-oic acid and bornyl cinnamate showing inhibitory effects against wood-decaying fungi, including *Lenzites betulina* and *Laetiporus sulphureus* [3]. In addition, the ethyl acetate fraction of *L. formosana* leaf extract has demonstrated antioxidant, hypoglycemic, anti-glycation, and antidiabetic properties [4]. Yao Zhu et al. further reported the chemical composition and antimicrobial activities of *L. formosana* oleoresins, identifying 15 pentacyclic triterpenoids, predominantly of the lupane and oleanane types, suggesting potential applications in the treatment of pathological angiogenesis [5].

Aging is a progressive pathophysiological process that leads to an irreversible decline in organ function. Numerous studies have shown that aging increases the risk of various common chronic diseases [6], including diabetes, neurodegenerative disease, cardiovascular disease, chronic obstructive pulmonary disease (COPD), and idiopathic pulmonary fibrosis (IPF). Cellular senescence, one of the hallmarks of aging, is a state of stable cell cycle arrest induced by diverse stressors such as DNA damage, oxidative stress, oncogene activation, and telomere shortening [7]. Although senescent cells lose their proliferative capacity, they remain metabolically active and acquire a pro-inflammatory phenotype known as the senescence-associated secretory phenotype (SASP), characterized by the secretion of cytokines, chemokines, proteases, and growth factors. While senescence plays beneficial roles in tumor suppression and tissue repair, its age-related accumulation promotes chronic inflammation and contributes to pathologies such as cancer, fibrosis, and neurodegeneration [8].

Consequently, targeting senescent cells has emerged as a promising therapeutic approach for age-associated diseases. Two main pharmacological strategies are currently under investigation: senolytics, which selectively eliminate senescent cells, and senomorphics, which modulate the senescent phenotype without inducing cell death, often by suppressing SASP factors or restoring cellular function [9]. Among the key biomarkers of cellular senescence, p16^INK4A^, a cyclin-dependent kinase inhibitor encoded by the CDKN2A gene, plays a critical role [10]. p16^INK4A^ functions as a tumor suppressor by inhibiting cyclin-dependent kinases CDK4 and CDK6, thereby blocking the phosphorylation of the retinoblastoma protein (pRB) and inducing cell cycle arrest in the G1 phase [11]. While p16^INK4A^ is essential for maintaining cellular homeostasis, its aberrant overexpression has been implicated in aging and age-related diseases. Elevated levels of p16^INK4A^ are frequently observed in aged tissues, where it promotes the accumulation of senescent cells and the activation of SASP secretion. Recent studies have further revealed that p16^INK4A^ functions not only as a biomarker of cellular senescence but also as an active driver of aging-related pathologies by impairing tissue regeneration and promoting degenerative processes.

Therefore, modulation of p16^INK4A^ expression using natural products or herbal medicines has emerged as a promising therapeutic strategy to delay senescence and promote healthy aging. Among the potential candidates, bakuchiol, a phenolic monoterpene isolated from *Psoralea corylifolia*, has been reported to exert a broad range of pharmacological activities, including significant anti-aging effects [12]. The roots of *L. formosana*, rich in phenolic monoterpenoids, represent another potential source of senescence-modulating agents. However, the anti-senescence potential of these compounds remains largely unexplored.

## 2. Materials and Methods

### 2.1. General Experimental Procedures

A JASCO P-2000 polarimeter (JASCO International Co. Ltd., Tokyo, Japan) was used to determine optical rotations and a Chirascan-Plus spectrometer (Applied Photophysics Ltd., Surrey, UK) was employed to record UV and electronic circular dichroism (ECD) spectra. IR spectra were measured using a Nicolet 6700 Fourier transform infrared spectrometer (Thermo Fisher Scientific, Waltham, MA, USA). 1D and 2D NMR spectra were acquired on a JEOL 400 MHz spectrometer, and data processing and assignments were performed with MestReNova 14.2 (Mestrelab Research, Santiago de Compostela, Spain). High-resolution electrospray ionization mass spectrometry (HR-ESI-MS) was performed on an Agilent Infinity UHPLC 1290 system equipped with an Agilent qTOF mass spectrometer 6530 (Agilent Technologies, Santa Clara, CA, USA). Compound purification was performed using a Gilson HPLC system with UV detection at 210 and 254 nm (Gilson Inc., Middleton, WI, USA), using a C18 column (10 × 250 mm, 10 μm particle size). Column chromatography (CC) was carried out with silica gel (particle size: 63–200 μm, Zeochem AG, Rüti, Switzerland), RP-C18 (particle size: 75 μm, Nacalai Tesque, Kyoto, Japan), and Sephadex LH-20 (GE Healthcare, Little Chalfont, UK). Thin-layer chromatography (TLC) was conducted using silica gel 60 F254 and RP-18 F254S plates (Merck, Darmstadt, Germany). All solvents were purchased from Daejung Chemical (Siheung, Republic of Korea).

### 2.2. Plant Material

The roots of *Liquidambar formosana* were collected in 2022 from the Gwangyang branch of the Seoul National University Forests (35°2′3.336″ N, 127°36′16.056″ E), Republic of Korea. Voucher specimen (SNU-2022-07) was deposited in the Herbarium of the College of Pharmacy, Seoul National University, Seoul, Republic of Korea. The plant was authenticated through integrated morphological and anatomical analyses by Hak-Ki Park from the Gwangyang branch of the Seoul National University Forests.

### 2.3. HR-ESI-MS/MS and Molecular Networking

HR-ESI-MS/MS analyses were performed on an Agilent 6530 Q-TOF mass spectrometer (Agilent Technologies, Santa Clara, CA, USA) interfaced with an Agilent 1260 Infinity UPLC and fitted with an ACQUITY UPLC BEH C18 column (2.1 × 100 mm i.d., 1.7 μm). The mobile phase gradient employed H_2_O with 0.1% formic acid (A) and MeCN (B), programmed from 10% B to 90% B over 20 min at 0.3 mL/min, maintained at 100% B for 4 min at 0.5 mL/min, returned to 10% B in 0.1 min, and equilibrated for 2 min at 0.3 mL/min. Nitrogen was used as both the drying and collision gas in the ESI source. The electrospray ionization parameters were optimized as follows: drying gas flow rate, 10 L/min; capillary temperature, 350 °C; sheath gas temperature, 350 °C; sheath gas flow, 12 L/min; nebulizer pressure, 30 psi. Additional source parameters included a capillary voltage (VCap) of 4000 V, fragmentor voltage of 180 V, skimmer voltage of 60 V, and an octopole RF peak voltage of 750 V. Data acquisition was performed in both positive and negative ionization modes with MS scanning from *m*/*z* 100–1000. Auto-MS/MS data were acquired in data-dependent mode with a fixed collision energy of 50 eV. ProteoWizard was employed to transform raw data files into mzML (Version 3.0, Palo Alto, CA, USA). Data processing was performed using MZmine software (version 4.0.3) through a workflow: mzML file import, peak threshold setting, chromatogram building, deconvolution, isotope grouping, join alignment, and gap filling. The processed data were exported as mgf-clustered spectral files and corresponding CSV metadata files (containing retention times, peak areas, and molecular formula assignments) using the integrated “Export for GNPS” and “Export to CSV file” functions. Molecular networking analysis was performed using the spectral/molecular networking module in MZmine. Library matching against the Global Natural Products Social Molecular Networking (GNPS) spectral databases required a minimum cosine score of 0.7 and at least four matched fragment ions. Network visualization and analysis were accomplished using Cytoscape software (version 3.10.3) (Figure S1).

### 2.4. Extraction and Isolation

The ground roots (3 kg) of *Liquidambar formosana* were extracted three times with 70% EtOH (5 L, 99 min each; Powersonic 420 ultrasonic cleaner, Hwashin Tech, Seoul, Republic of Korea) at room temperature to obtain a crude extract, which was then dried under reduced pressure. The dried crude root extract was partitioned between n-hexane, ethyl acetate, n-butanol, and water to obtain four respective fractions. The hexane fraction was chromatographed using an MPLC system (Biotage, Uppsala, Sweden) equipped with an RP-C18 column (40–60 µm particle size, MeOH/H_2_O = 10/90 to 100/0), yielding three fractions (M1 to M3). Fraction M2 (2 g) was subjected to MPLC on a normal-phase (NP) column and eluted with n-hexane/EtOAc (85/15 to 100/0), yielding three subfractions. Fraction M2.1 (1.5 g) was further chromatographed on an RP column (40–60 µm particle size; MeOH/H_2_O = 80/20 to 100/0) to obtain three subfractions. Fraction M2.1.3 (500 mg) was purified by semi-preparative HPLC (MeCN/H_2_O, isocratic 90%) to yield compound **7** (100 mg, 23 min), compound **6** (32 mg, 21.5 min), compound **3** (30 mg, 17 min), compound **4** (4 mg, 15 min), compound **5** (7 mg, 12.5 min), compound **2** (8 mg, 16.5 min), compound **1** (4 mg, 20.5 min), and compound **8** (2 mg, 13.5 min).

β-Pinenyl cinnamate (**1**): Pale yellow amorphous powder; ${\left[\alpha \right]}_{D}^{25}$ + 99.6 (*c* 0.1, MeOH); IR (KBr) *ν*_max_ 3898, 3730, 3564, 2930, 2353, 1711, 1636, 1513, 1452, 1278, 1166, 981, 769, 684; ^1^H and ^13^C NMR; see Table 1 and Figures S2–S10 in Supplementary Materials; HRESIMS *m*/*z* 283.1707 [M + H]^+^ (calcd for C_19_H_23_O_2_ at 283.1693, *m*/*z* error 4.9 ppm).

Myrcenyl cinnamate (**2**): Pale yellow amorphous powder; ${\left[\alpha \right]}_{D}^{25}$ + 107.8 (*c* 0.1, MeOH); UV (MeOH) *λ*_max_ (log *ε*) 252 (−1.95), 350 (−1.85); IR (KBr) *ν*_max_ 3898, 3730, 3564, 2930, 2353, 1711, 1636, 1513, 1452, 1278, 1166, 981, 769, 684; ^1^H and ^13^C NMR; see Table 1 and Figures S11–S17; HRESIMS found *m*/*z* 283.1694 [M + H]^+^ (calcd for C_19_H_23_O_2_ at 283.1693, *m*/*z* error 0.3 ppm)

Limonenyl cinnamate (**3**): Pale yellow amorphous powder; ^1^H NMR (400 MHz, CDCl_3_) δ_H_ 7.68 (dd, *J* = 16.0, 4.5 Hz, 1H), 7.58–7.48 (m, 2H), 7.44–7.34 (m, 3H), 6.46 (dd, *J* = 16.0, 11.6 Hz, 1H), 5.62 (tp, *J* = 2.8, 1.4 Hz, 1H), 4.59 (s, 2H), 2.43 (dt, *J* = 8.6, 5.6 Hz, 1H), 2.31 (dt, *J* = 15.2, 1.6 Hz, 1H), 2.18 (td, *J* = 5.6, 1.5 Hz, 1H), 2.12 (dqd, *J* = 5.9, 2.9, 1.2 Hz, 1H), 1.30 (s, 3H), 1.22 (d, *J* = 8.7 Hz, 1H), 0.86 (s, 3H); ^13^C NMR (100 MHz, CDCl_3_) δ_C_ 167.0, 144.8, 143.2, 134.6, 130.4, 128.2, 128.2, 121.6, 118.3, 67.2, 43.7, 40.8, 38.2, 31.6, 31.4, 29.8, 26.2, 21.2.; HRESIMS *m*/*z* 283.1706 [M + H]^+^ (calcd for C_19_H_23_O_2_ at 283.1693, *m*/*z* error 4.6 ppm).

4-Isopropylbenzyl cinnamate (**4**): Pale yellow amorphous powder; ^1^H NMR (400 MHz, CDCl_3_) δ_H_ δ 7.72 (d, *J* = 16.0 Hz, 1H), 7.55–7.48 (m, 2H), 7.41–7.32 (m, 5H), 7.25 (d, *J* = 5.2 Hz, 2H), 6.48 (d, *J* = 16.0 Hz, 1H), 5.22 (s, 2H), 1.29–1.22 (m, 6H); ^13^C NMR (100 MHz, CDCl_3_) δ_C_ 167.0, 149.3, 145.2, 133.5, 130.5, 129.0, 128.7, 128.2, 126.8, 118.1, 66.5, 34.1, 24.1; HRESIMS *m*/*z* 281.1556 [M + H]^+^ (calcd for C_19_H_23_O_2_ at 281.1536, *m*/*z* error 7.11 ppm).

Cinnamyl cinnamate (**5**): Pale yellow amorphous powder; ^1^H NMR (400 MHz, CDCl_3_) δ_H_ 7.74 (d, *J* = 16.0 Hz, 1H), 7.54 (dt, *J* = 6.3, 3.8 Hz, 3H), 7.45–7.35 (m, 6H), 7.34–7.31 (m, 2H), 6.53–6.43 (m, 1H), 4.88 (dd, *J* = 6.4, 1.3 Hz, 2H); ^13^C NMR (100 MHz, CDCl_3_) δ_C_ 166.9, 145.3, 134.5, 134.4, 130.5, 129.1, 128.8, 128.3, 128.2, 126.8, 123.4, 118.1, 65.3, 29.9.

Myrthenyl cinnamate (**6**): Pale yellow amorphous powder; ^1^H NMR (400 MHz, CDCl_3_) δ_H_ 7.68 (d, *J* = 16.0 Hz, 1H), 7.54 (dq, *J* = 5.7, 3.2 Hz, 2H), 7.41–7.36 (m, 3H), 6.48 (d, *J* = 16.0 Hz, 1H), 5.02 (ddd, *J* = 10.0, 3.5, 2.1 Hz, 1H), 2.42 (ddt, *J* = 13.9, 10.0, 4.0 Hz, 1H), 2.05 (ddd, *J* = 12.7, 9.1, 4.4 Hz, 1H), 1.85–1.68 (m, 2H), 1.42–1.23 (m, 3H), 1.06 (dd, *J* = 13.8, 3.5 Hz, 1H), 0.95 (s, 3H), 0.90 (s, 3H), 0.89 (s, 3H); ^13^C NMR (100 MHz, CDCl_3_) δ_C_ 167.1, 144.9, 143.2, 134.6, 130.4, 129.0, 128.2, 128.2, 121.7, 118.3, 67.3, 43.8, 40.9, 38.2, 31.6, 31.4, 29.8, 26.3, 21.2; HRESIMS *m*/*z* 283.1691 [M + H]^+^ (calcd for C_19_H_23_O_2_ at 283.1693, *m*/*z* error −0.71 ppm).

Bornyl cinnamate (**7**): Pale yellow amorphous powder; ^1^H NMR (400 MHz, CDCl_3_) δ_H_ 7.68 (d, *J* = 16.0 Hz, 1H), 7.54 (dq, *J* = 5.7, 3.2 Hz, 2H), 7.41–7.36 (m, 3H), 6.48 (d, *J* = 16.0 Hz, 1H), 5.02 (ddd, *J* = 10.0, 3.5, 2.1 Hz, 1H), 2.42 (ddt, *J* = 13.9, 10.0, 4.0 Hz, 1H), 2.05 (ddd, *J* = 12.7, 9.1, 4.4 Hz, 1H), 1.85–1.68 (m, 2H), 1.42–1.23 (m, 3H), 1.06 (dd, *J* = 13.8, 3.5 Hz, 1H), 0.95 (s, 3H), 0.90 (s, 3H), 0.89 (s, 3H); ^13^C NMR (100 MHz, CDCl_3_) δ_C_ 167.5, 144.4, 134.7, 130.3, 129.0, 128.2, 119.0, 80.1, 49.1, 48.0, 45.1, 37.0, 29.8, 28.2, 27.4, 19.9, 19.0, 13.7; HRESIMS *m*/*z* 285.1846 [M + H]^+^ (calcd for C_19_H_25_O_2_ at 285.1849, *m*/*z* error −1.07 ppm).

3-Phenylpropyl cinnamate (**8**): Pale yellow amorphous powder; ^1^H NMR (400 MHz, CDCl_3_) δ_H_ 7.68 (d, *J* = 16.0 Hz, 1H), 7.57–7.51 (m, 2H), 7.40 (dt, *J* = 4.6, 2.7 Hz, 3H), 7.33–7.27 (m, 2H), 7.24–7.17 (m, 3H), 6.45 (d, *J* = 16.0 Hz, 1H), 4.24 (t, *J* = 6.5 Hz, 2H), 2.75 (dd, *J* = 8.6, 6.8 Hz, 2H), 2.13–1.99 (m, 2H); ^13^C NMR (100 MHz, CDCl_3_) δ_C_ 167.2, 144.9, 141.4, 134.6, 130.4, 129.0, 128.6, 128.2, 126.2, 118.3, 64.1, 32.4, 30.5.; HRESIMS *m*/*z* 267.1375 [M + H]^+^ (calcd for C_19_H_23_O_2_ at 267.1380, *m*/*z* error −1.87 ppm).

### 2.5. ECD Calculation

A conformational search was performed using the GMMX extension in Gaussian View 9 with MMFF94s molecular force-field calculations and an energy cutoff of 3.5 kcal/mol. Conformers with populations greater than 1% were selected and then subjected to DFT optimization followed by frequency calculations at the B3LYP/6-31G(d) level of theory in the gas phase using Gaussian 16. Upon completion, Gibbs free energies were extracted, and Boltzmann populations were calculated. Conformers with a Boltzmann population greater than 1% were selected for ECD simulation using time-dependent DFT (TD-DFT) at the B3LYP/6-31G(d) level of theory in MeOH (IEFPCM solvent model), considering up to the first 20 excitation states (compound **1**). The Boltzmann-averaged spectra were visualized using SpecDis 1.70 software, with a half-bandwidth correction of 0.2–0.3 eV and a UV shift correction of 0–20 nm.

### 2.6. Cell Culture

The lung cancer cell line A549 was obtained from the Korean Cell Line Bank. A549 cells were cultured in Roswell Park Memorial Institute (RPMI) 1640 medium (Welgene, Daegu, Republic of Korea) supplemented with 10% fetal bovine serum (FBS) (Gibco, Waltham, MA, USA) and 1% penicillin-streptomycin (P/S; Gibco) under a humidified atmosphere containing 5% CO_2_ at 37 °C. IMR90 human lung fibroblasts (American Type Culture Collection, ATCC, CCL-186) were maintained in Minimum Essential Medium (MEM) supplied with 10% FBS (Gibco) and 1% P/S at 37 °C with 5% CO_2_. Cells were subcultured every 48 hr.

### 2.7. p16^INK4A^ Promoter Activity-Related Luciferase Reporter Assay

A549 cells were seeded at a density of 80,000 cells/mL in 96-well plates. After 24 hr of incubation, they were washed with phosphate-buffered saline (PBS) and transfected with a plasmid mixture and Lipofectamine 2000 (Invitrogen, Carlsbad, CA, USA) for 7 hr in Opti-MEM. The plasmid mixture contained 0.07 μg pGL3_p16^INK4A^ plasmid encoding the human p16^INK4A^ promoter (GenBank accession no. NM_000077.4) and firefly luciferase, and 0.03 μg pSV-beta-galactosidase plasmid (Promega, Madison, WI, USA) as an internal standard. The following day, cells were treated with compounds and navitoclax (as a positive control) for 20 h, followed by lysis with cell culture lysis buffer (Promega). Luciferase activity was measured using a firefly luciferase assay kit (Promega) and normalized to β-galactosidase activity.

### 2.8. MTT Assay

Cell viability was assessed by the (3-(4,5-dimethyl-2-thiazolyl)-2,5-diphenyl-2H-tetrazolium bromide (MTT; Sigma, St. Louis, MO, USA) assay. A volume of 20 μL of the 2 mg/mL MTT solution was added to each well in a 96-well plate, and the plates were incubated at 37 °C in the dark for 4 h. After incubation, the supernatant was removed, and the formazan crystals were dissolved in 100 μL of DMSO. The absorbance was obtained at 540 nm using a microplate reader (VersaMax^TM^, Molecular Devices, San Jose, CA, USA).

### 2.9. Senescence-Associated β-Galactosidase Staining

IMR90 cells were seeded at 50,000 cells/mL in 24-well plates and incubated for 24 h. Bleomycin (40 µg/mL; Tokyo Chemical Industry, Tokyo, Japan) was added to all wells except the untreated control. After 48 h, the test compounds were introduced and cells were further incubated for 48 h. Cells were stained using a senescence-associated β-galactosidase (SA-β-gal) staining kit (Cell Signaling Technology, Danvers, MA, USA) according to the manufacturer’s instructions. The stained cells were observed under a microscope (Olympus Corporation, Tokyo, Japan). All reactions were independently performed in triplicate. The expression level of X-gal was normalized to that of the control group.

### 2.10. RNA Extraction and Quantitative Real-Time Polymerase Chain Reaction (qRT-PCR)

IMR90 cells were seeded as described above and treated with bleomycin (40 µg/mL) for 48 h, followed by exposure to the test compounds for an additional 48 h. Total RNA was extracted using NucleoZOL (Macherey-Nagel, Düren, Germany) after 48 h of compound treatment. One µg of total RNA was used for cDNA synthesis with the Thermo cDNA Synthesis Kit (Thermo Fisher Scientific). Real-time qPCR was performed by the AccuPower 2X GreenStarTM qPCR Master Mix (Bioneer, Daejeon, Republic of Korea). All reactions were performed in independent triplicates. The expression levels of all markers were normalized to 18s ribosomal RNA.

### 2.11. Statistical Analyses

The data were analyzed using GraphPad PRISM 10.4.1 software (GraphPad Software, Inc., La Jolla, San Diego, CA, USA) and are expressed as the mean ± standard error of the mean (SEM) from three independent experiments. To compare group means, analysis of variance (ANOVA) was performed, followed by Dunn’s multiple comparison test for post hoc analysis. Statistical significance was established at * *p* < 0.05, ** *p* < 0.01, *** *p* < 0.001, and **** *p* < 0.0001.

## 3. Results

### 3.1. Feature-Based Molecular Networking of the Liquidambar formosana Root Extract

The GNPS molecular network highlighted six noteworthy clusters, including coumarins, gallotannins, monoterpenoids, fatty acids, triterpenoids, and flavonoids. In the triterpenoid cluster, four compounds were annotated using the GNPS platform against the online GNPS database, namely, betulonic acid, sumaresinolic acid, ursolic acid, and oleanolic acid. The largest cluster displayed the presence of numerous compound classes, including monoterpenoids such as (−)-carveol (*m*/*z*: 135.1170) and α-pinene (*m*/*z*: 137.1320), along with coumarins and fatty acids.

To enhance the annotation of metabolites from *L. formosana* roots, a local database was established by searching Reaxys for compounds previously isolated from *L. formosana*. This local database was then imported into MZmine to calculate the possible adducts and annotate features by matching with the corresponding *m*/*z* values. The result showed that many nodes were annotated, including a monoterpene cluster [linalool (*m*/*z* 137.1320)]; a gallotannin cluster [3,4,5-trimethoxyphenyl-6-O-syringoyl-D-glucopyranoside (*m*/*z* 527.1729) and 2,4,6-tri-O-galloyl-D-glucose (*m*/*z* 619.0930)]; and a triterpene cluster [melliferone (*m*/*z* 435.3257) and 28-diepoxyoleanane-3-one (*m*/*z* 437.3414)]. Catechin (*m*/*z* 273.0757) and gallocatechin (*m*/*z* 289.0707) were also putatively identified. However, a significant number of molecular features remained unannotated, which represents a well-known challenge in metabolomic analysis, where many detected chemical signals cannot be identified using standard library matching. To address this bottleneck, we applied the Structural similarity Network Annotation Platform for Mass Spectrometry (SNAP-MS). This tool is designed to annotate molecular networking subnetworks without relying on experimental MS^2^ spectral libraries, instead leveraging the principle that the distribution of molecular formulae within a natural product family is often highly diagnostic. For each subnetwork, SNAP-MS searches each parent mass against a comprehensive reference database, in this instance, the COCONUT database, which contains over 600,000 compounds from natural sources. It retrieves all potential candidate structures for each mass and then groups them into compound families based on structural similarity. These families are scored and ranked according to their coverage, defined as the number of distinct masses from the original subnetwork represented within that family. This approach enables more accurate prediction of a compound family for an entire subnetwork, even when individual members are absent from spectral libraries [13].

**Figure 1:**
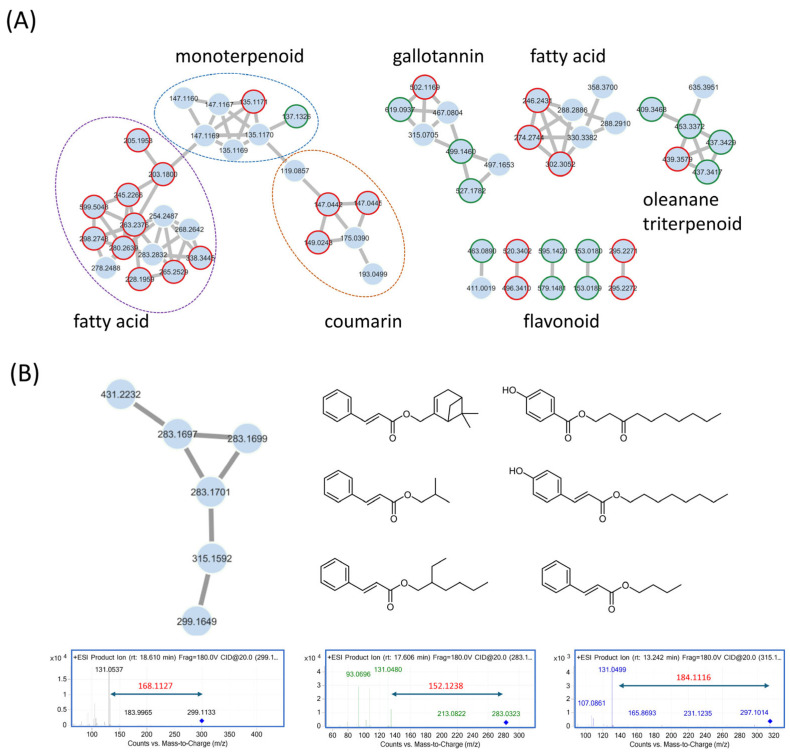
(**A**) Clusters of interest from molecular networking of the crude extract from the roots of *L. formosana* using UPLC qTOF-MS/MS data acquired in the positive mode. Nodes were grouped into chemical classes, and clusters were circled as follows: blue for monoterpenoids, purple for fatty acids, and orange for coumarins. Individual nodes annotated by GNPS are highlighted with a red ring, while nodes putatively identified from the literature (via a Reaxys search for compounds previously reported from *L. formosana*) are marked with a green ring. (**B**) The target cluster for isolation (left) and suggested structures for cluster (right), as revealed by SNAP-MS and tandem MS analyses, show cinnamic acid derivatives bearing possible monoterpene moieties.

Results from SNAP-MS suggested six clusters with top candidates, including xanthones, monoterpenoids, diarylheptanoids, stilbenoids, coumarins, and cinnamic acid-conjugated monoterpenoids (Figure S18). Among them, the cluster of cinnamic acid-conjugated monoterpenoids is described in Figure 1B. MS/MS analysis revealed a characteristic fragment at *m*/*z* 131.05. This fragment corresponds to the protonated cinnamic acid moiety, providing strong experimental evidence for the presence of a cinnamate substructure across the molecular family. Using this ion as a marker, we extracted the ion chromatogram (± 0.05 Da) of the enriched fraction and observed several peaks whose MS^2^ spectra contained *m*/*z* 131.05. The corresponding precursor ions were *m*/*z* 265, 267, 281, 283, and 285, consistent with multiple monoterpene–cinnamate conjugates. This combination of computational prediction from SNAP-MS and direct experimental evidence from the MS/MS data provided valuable guidance for the subsequent targeted isolation of these novel compounds.

### 3.2. Isolation and Structural Elucidation of New Compounds ***1*** and ***2***

**Figure 2:**
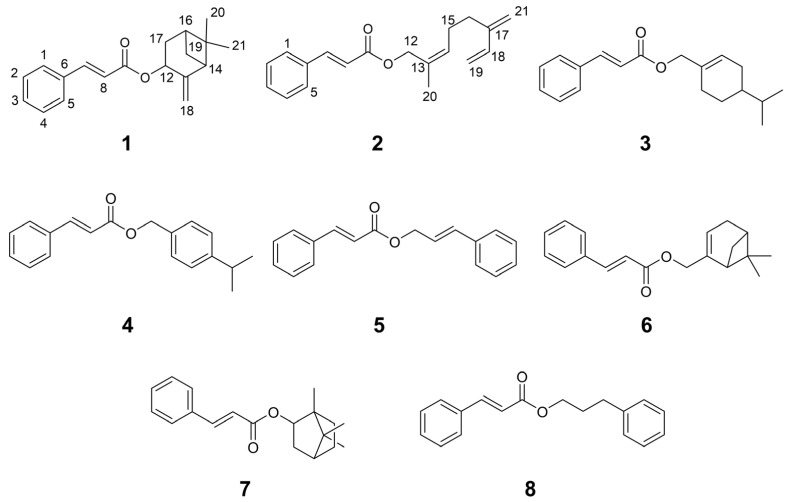
Chemical structures of eight compounds isolated from the roots of *L. formosana*. Using a bioactivity-guided isolation strategy, the 70% EtOH root extract was partitioned with n-hexane, followed by MPLC and HPLC. The chemical structures of each compound were elucidated based on 1D and 2D NMR, IR, and high-resolution mass spectrometry. Compounds **1** and **2** were identified as newly discovered natural products.

**Figure 3:**
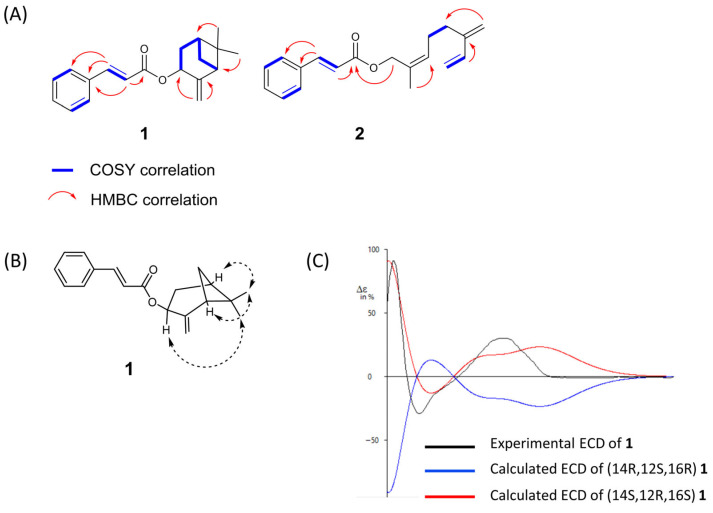
(**A**) Key COSY (blue bold lines) and HMBC (red arrows) correlations for compounds **1** and **2**. (**B**) Key NOE correlations for compound **1** (dashed-double-headed arrows), with the relative configuration assigned based on NOE data. (**C**) Experimental and calculated ECD spectra of **1**. Conformers with populations greater than 1% were optimized and frequency-verified by DFT [B3LYP/6-31G(d), gas phase; Gaussian 16]. The experimental ECD (black) was in good agreement with the calculated spectrum (red), establishing the absolute configuration as 14S,12R,16S.

The structure of compound **1** was deduced using ^1^H and ^13^C NMR, one- and two-dimensional spectroscopy, as well as IR and MS analyses (Figure 2). *UPLC* qTOF-MS/MS analysis of **1** yielded a protonated molecular ion [M + H]^+^ at *m*/*z* 283.1710 (calcd for C_19_H_23_O_2_, 283.1693), consistent with a molecular formula of C_19_H_22_O_2_, indicating nine degrees of unsaturation. The ^1^H NMR spectrum exhibited characteristic signals of a trans-disubstituted double bond at δ_H_ 7.68 and 6.45 (each d, *J* = 16.0 Hz), an oxygenated methine at δ_H_ 5.70, two protons of an exomethylene group (δ_H_ 5.11, 4.93), and a vinyl methyl group at δ_H_ 0.73 and 1.31 (each s). The ^13^C NMR data, in conjunction with DEPT experiments, revealed 19 resonances attributable to one carbonyl (δ_C_ 166.7), six aromatic carbons (δ_C_ 128.2, 128.2, 129.0, 129.0, 130.3, 134.6), four olefinic carbons (δ_C_ 144.6, 119.04, 150.5, 114.4), two quaternary sp3 carbons (δ_C_ 40.7, 150.5), two methylenes (δ_C_ 28.1, 33.5) including one exomethylene (δ_C_ 114.4), and two methyl carbons (δ_C_ 22.1, 26.0). COSY, HSQC, and HMBC spectral data provided additional evidence to confirm the structure of compound **1**. The COSY spectrum of compound **1** displayed connectivities between H-16 (δ_H_ 2.02, m) and H-17 (δ_H_ 1.88, 2.45, each m), H-16 and H-19 (δ_H_ 1.58, 1.69, each m), H-19 and H-14 (δ_H_ 2.56, t, *J* = 5.4 Hz), and H-17 and H-12 (δ_H_ 5.70, d, *J* = 8.0 Hz) (Figure 3A). The HMBC spectrum of compound **1** showed significant correlations from the exomethylene protons (δ_H_ 4.93, 5.11, each br s, H-18) to C-13 (δ_C_ 150.5), C-12 (δ_C_ 68.8), and C-14 (δ_C_ 50.9). The signals for H-19 correlated with C-13, C-14, C-15 (δ_C_ 40.7), C-16 (δ_C_ 39.7), and C-17 (δ_C_ 33.5), whereas H-20 (δ_H_ 0.73) and H-21 (δ_H_ 1.31) showed correlations with C-15 and C-21 (δ_C_ 26.0). In addition, the correlations of H-7 (δ_H_ 7.68, d, *J* = 16.0 Hz) with C-5 (δ_C_ 134.6), C-6 (δ_C_ 128.2), and C-4 (δ_C_ 128.2), as well as the correlation of H-8 (δ_H_ 6.45, d, *J* = 16.0 Hz) with C-5, supported the proposed structure as a cinnamic acid conjugated with β-pinene. The relative configuration of **1** was determined based on diagnostic NOE correlations (Figure 3B). NOE correlations between Me-20 and H-14, and between Me-20 and H-16, revealed an α-orientation, whereas the NOE correlation between Me-21 and H-12 indicated a β-orientation. Based on the relative configuration, the absolute configuration of **1** was assigned as 14S,12R,16S by ECD calculations (Figure 3C). Therefore, compound **1** was characterized as β-pinenyl cinnamate.

The structure of compound **2** was elucidated through comprehensive analysis of ^1^H and ^13^C NMR, one and two-dimensional spectroscopic techniques, as well as IR and MS data. HR-ESI-MS analysis of **2** showed a protonated molecular ion [M + H]^+^ at *m*/*z* 283.1715, consistent with the molecular formula of C_19_H_22_O_2_ (calcd *m*/*z* 283.1693), indicating nine degrees of unsaturation. The ^1^H NMR spectrum exhibited characteristic signals of a trans-disubstituted double bond at δ_H_ 7.70 and 6.47 (each d, *J* = 16.0 Hz), an oxygenated methylene at δ_H_ 4.60 (s, 2H), and a vinyl methyl group at *δ*_H_ 1.71 (s). The ^13^C NMR spectrum, in conjunction with DEPT experiments, revealed 19 resonances, corresponding to one carbonyl carbon (δ_C_ 167.0), six aromatic carbons (δ_C_ 129.0, 129.0, 128.2, 128.2, 130.4, 134.5), eight olefinic carbons (δ_C_ 145.8, 144.9, 138.9, 130.7, 129.3, 118.2, 116.1, 113.3), three methylenes (δ_C_ 26.5, 30.9, 70.3), two exomethylenes (δ_C_ 113.3, 116.1), and one methyl carbon (δ_C_ 14.2). The COSY spectrum of **2** revealed key connectivities between H-19 [(δ_H_ 5.07, d, *J* = 10.8 Hz), (δ_H_ 5.24, d, *J* = 17.6 Hz)] and H-18 (δ_H_ 6.38, dd, *J* = 17.6, 10.8 Hz), and H-16 (δ_H_ 2.28, br s) with H-15 (δ_H_ 2.26, br s) and H-14 (δ_H_ 7.38, m), confirming the presence of a myrcene moiety (Figure 3A). HMBC correlations from H-7 (δ_H_ 7.70, d, *J* = 16.0 Hz) to C-5 (δ_C_ 134.5), C-6 (δ_C_ 128.2), and C-4 (δ_C_ 128.2), as well as from H-8 (δ_H_ 6.47, d, *J* = 16.0 Hz) to C-5 (δ_C_ 134.5), supported the cinnamic acid substructure. In addition, HMBC correlations from the oxymethylene protons (δ_H_ 4.60, H-12) to the carbonyl carbon (δ_C_ 167.0, C-9) established the ester linkage between the cinnamic acid and myrcene unit. Therefore, compound **2** was characterized as myrcenyl cinnamate.

Comparison with previously reported literature allowed us to identify compounds **3**–**8** as limonenyl cinnamate (**3**) [14], 4-isopropylbenzyl cinnamate (**4**) [15], cinnamyl cinnamate (**5**) [16], myrthenyl cinnamate (**6**) [17], bornyl cinnamate (**7**) [18], and 3-phenylpropyl cinnamate (**8**) [19].

### 3.3. Bioactivity of Isolated Compounds from Liquidambar formosana

Cellular senescence, as described by the Hayflick limit, was first demonstrated by the cessation of cell growth after several passages in cell culture [20]. A key regulator of this process is p16^INK4A^, which initiates and maintains senescence by binding to CDK4/6, thereby inhibiting its kinase activity and preventing phosphorylation of Rb during the G1-to-S transition in order to regulate the cell cycle [21]. Several studies have revealed that p16^INK4A^ expression is significantly increased in senescent cells during natural aging and in age-related diseases [22]. Recent studies have highlighted phenolic compounds, such as cinnamic acid derivatives, for their anti-senescence potential. Kanlayavattanakul et al. showed that hydroxycinnamic acids can alleviate aging in dermal cells [23]. Caffeic acid (3,4-dihydroxycinnamic acid), a plant-derived phenolic antioxidant, has been reported to inhibit skin aging in human keratinocytes (HaCaT cells) [24] and in skin cancer xenograft mouse models [25]. Along with cinnamic acid, numerous studies on the senomorphic effects of monoterpenes have demonstrated their ability to inhibit SASP by modulating key signaling pathways such as NF-κB, mTOR, and JAK/STAT [26]. β-myrcene has been reported to exhibit senomorphic activity, particularly through the direct suppression of IL-6 secretion in human dermal fibroblast cells [27]. Recently, the phenolic monoterpene bakuchiol has been gaining attention for its anti-aging effects [24]. However, few other phenolic monoterpenes or cinnamic acid-conjugated monoterpenes have been identified as anti-aging agents.

**Figure 4:**
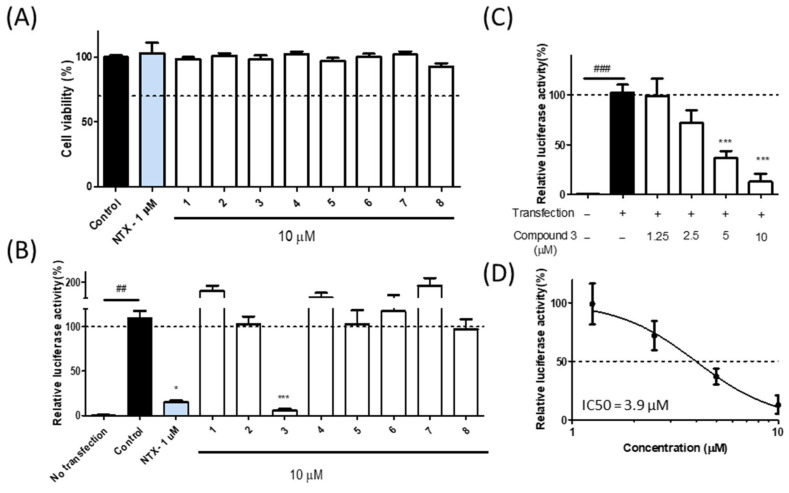
Effect of compounds on inhibition of the p16^INK4A^ promoter activity. (**A**) Cell viability of compounds **1**–**8** from *L. formosana* was measured using an MTT assay. Cell viability results are expressed as relative values compared with the untreated group (set at 100%). Bar colors indicate groups: black, control; blue, navitoclax treated control; white, experimental. (**B**) Inhibitory effects of compounds **1**–**8** on p16^INK4A^ promoter activity. Bar colors indicate groups as in panel (**A**). (**C**,**D**) Inhibitory effect of compound **3** in a dose-dependent manner (1.25, 2.5, 5, and 10 µM). (**C**) Compound **3** reduced p16^INK4A^ promoter activity (black, control; white, treated. (**D**) Dose–response curve of compound **3** based on data from panel (**C**). Data were normalized to β-galactosidase activity and are presented as mean ± SEM (*n* = 3). Statistical analysis was performed using one-way ANOVA followed by Dunn’s multiple comparisons test (* *p* < 0.05, *** *p* < 0.001 vs. control; ## *p* < 0.01, ### *p* < 0.001 vs. no transfection control).

In this study, eight isolated compounds (**1**–**8**) from the ethyl acetate fraction of *L. formosana* were assessed for cytotoxicity. All compounds exhibited over 70% cell viability at a concentration of 10 μM in an MTT cell viability assay (Figure 4A). To evaluate their effect on p16^INK4A^ expression, human lung carcinoma A549 cells, which have a homozygous deletion of p16^INK4A^ gene, were used [28]. A549 cells were co-transfected with p16^INK4A^ promoter-driven luciferase construct and β-galactosidase as an internal standard. Since the expression of p16^INK4A^ is regulated by its promoter, quantifying its transcriptional activity provides a suitable model for assessing this key aging biomarker. As a result of compound treatment at 10 µM, compound **3** significantly reduced luciferase activity driven by the p16^INK4A^ promoter, with an IC_50_ value of 3.9 µM. (Figure 4B–D). Navitoclax, a known senolytic agent, was used as a positive control to determine the downregulation of p16^INK4A^, with normalization by β–galactosidase.

**Figure 5:**
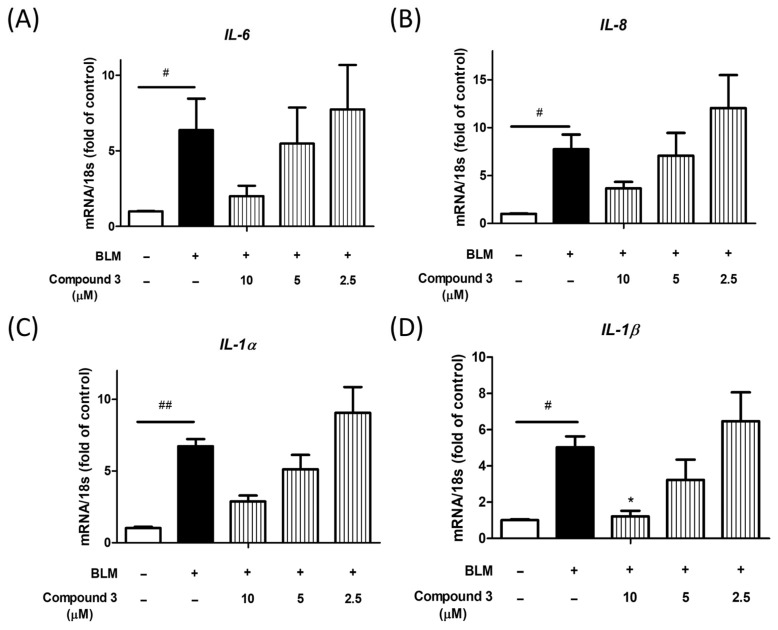
Senomorphic effects of compound **3** on rescuing replicative senescence and inhibiting SASP mRNA expression. IMR90 cells were exposed to bleomycin (BLM, 40 µg/mL) for 2 days and subsequently treated with the indicated compounds for an additional 2 days, after which total RNA was extracted for analysis. Compound **3** was tested in a dose-dependent manner (2.5, 5, and 10 µM) for its effects on SASP markers IL-6 (**A**), IL-8 (**B**), IL-1α (**C**), and IL-1β (**D**). Data are expressed as mean ± SEM (*n* = 3). Bar shading and pattern denote the following groups: white (control), black (bleomycin-only control), and striped (experimental). Statistical analysis was performed using one-way ANOVA followed by Dunn’s multiple comparisons test (* *p* < 0.05 vs. BLM-treated group for 2 days; # *p* < 0.05, ## *p* < 0.01 vs. control).

SASP, or senescence-messaging secretome (SMS), includes cytokines such as IL-6, IL-8, IL-1α, and IL-1β, which are characteristic features of senescent cells [29]. These cytokines not only serve as biomarkers but also play functional roles in reinforcing the senescent state and affecting surrounding tissues. For instance, interleukin-6 (IL-6) can induce premature senescence through activation of signal transducer and activator of transcription 3 (STAT3) signaling [30]. IL-8 is similarly overexpressed in senescent fibroblasts via autocrine signaling through the IL-8 pathway [31]. Besides IL-6 and IL-8, IL-1 family members, including membrane-bound IL-1α and soluble IL-1β, have also been studied as markers of senescence. Given this, we employed the bleomycin-induced senescence model in IMR90 fibroblasts, a well-established system for cellular aging studies, which leads to changes in the mRNA levels of SASP factors [32,33,34]. In this experiment, senescence was induced in IMR90 fibroblasts by a 2-day bleomycin pretreatment, followed by exposure to compound **3** for 48 h. The expression of four major SASP-associated genes (IL-6, IL-8, IL-1α, and IL-1β) was reduced in a dose-dependent manner upon treatment with compound **3** (Figure 5). At 10 µM, compound **3** markedly suppressed the expression of all four cytokines compared with the bleomycin-only control, with reductions of 68.55% for IL-6, 52.75% for IL-8, 57.18% for IL-1α, and 75.83% for IL-1β. At 5 µM, partial reductions were observed for IL-1α and IL-1β, whereas IL-6 and IL-8 remained largely unchanged. In contrast, treatment with 2.5 µM compound **3** did not produce significant effects on any of the tested markers. These findings indicate that compound **3** exerts the most robust and consistent inhibitory effects on SASP gene expression at 10 µM.

**Figure 6:**
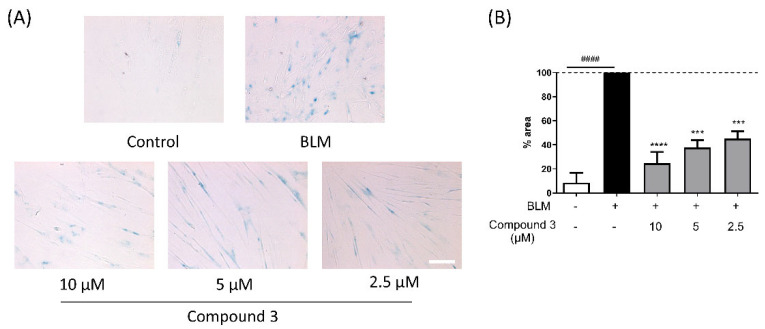
Results of the senescence-associated β-galactosidase (SA-β-gal) staining (**A**) and quantification. Bar shading denotes the following groups: white (control), black (bleomycin-only control), and gray (experimental). (**B**). IMR90 cells were exposed to bleomycin (BLM, 40 µg/mL) for 2 days and subsequently treated with the indicated compounds for an additional 2 days. Cells were then analyzed by SA-β-gal staining. Compound **3** was tested in a dose-dependent manner (2.5, 5, and 10 µM). The proportion of SA-β-gal-positive cells was significantly reduced in the compound **3**-treated groups compared with the bleomycin-only control. Data are presented as mean ± SEM (*n* = 3). Statistical analysis was determined using one-way ANOVA followed by Dunn’s multiple comparisons test (*** *p* < 0.001, **** *p* < 0.0001 vs. 2-day BLM-treated group; #### *p* < 0.0001 vs. control). Scale bars = 200 µm.

Senescence cell morphology typically displays an enlarged, flattened shape, together with molecular features such as increased p16^INK4A^ expression and reduced Rb phosphorylation. SA-β-gal is a well-established biomarker for the detection of the senescent phenotype. SA-β-gal staining is based on the detection of lysosomal β-galactosidase, which cleaves X-gal to produce a visible blue precipitate at pH 6.0 [12]. To validate the senomorphic activity of compound **3**, SA-β-gal staining was performed on bleomycin-induced senescent IMR90 fibroblasts. As shown in Figure 6A, cells exposed to bleomycin for 4 days displayed a pronounced increase in SA-β-gal-positive staining, characterized by enlarged and flattened morphology with extensive blue precipitates, compared with the untreated control group. In contrast, treatment with compound **3** for 48 h following bleomycin pretreatment markedly reduced the proportion of SA-β-gal-positive cells in a concentration-dependent manner. At 10 µM, treatment of compound **3** led to a marked reduction in SA-β-gal staining, whereas cells treated with 5 µM and 2.5 µM showed a moderate decrease compared with the bleomycin-only group. Quantitative analysis confirmed these observations (Figure 6B). Bleomycin pretreatment increased the proportion of SA-β-gal-positive area to nearly 100% relative to the control, whereas subsequent treatment with compound **3** reduced this value to 41.82 ± 26.72% at 10 µM, 43.07 ± 12.36% at 5 µM, and 46.84 ± 10.87% at 2.5 µM. These results demonstrate that compound **3** significantly attenuates bleomycin-induced cellular senescence, with the strongest inhibitory effect observed at the highest tested concentration (10 µM).

## 4. Discussion

In this study, two new compounds (**1** and **2**), along with six known compounds, were isolated from the root of *L. formosana* based on a bioactivity-guided isolation approach (Supplementary Figure S20). With the increasing use of high-resolution mass spectrometry, FBMN has been proven to be an efficient and reliable method for dereplication and chemical profiling of natural products due to the availability of ever-growing public databases. By using this method, two new compounds were isolated, thereby enhancing the chemical diversity of this plant.

Since its discovery, p16^INK4A^ has been shown to have a strong correlation with aging and cellular senescence [35]. Previous studies have demonstrated that ionizing radiation can induce senescence via the ROS-mediated p16^INK4A^ pathway in human vascular smooth muscle cells [36] and mouse hippocampus cells [37]. In this study, we initially performed a p16^INK4A^ promoter activity inhibition screening assay to identify bioactive compounds that inhibit p16^INK4A^ expression, a major biomarker of senescence. Using A549 cells, which have a homozygous deletion of the p16^INK4A^ gene [28], and co-transfected with the p16^INK4A^ gene and β-galactosidase as an internal standard, compound **3** was identified as a potent p16^INK4A^ inhibitor with an IC_50_ of 3.9 µM. SASP expression is regulated through multiple transcriptional and signaling pathways, with NF-κB identified as a master regulator [38]. In senescent cells, damaged DNA released into the cytoplasm forms cytoplasmic chromatin fragments (CCFs) that activate the cGAS–STING–NF-κB pathway, leading to the secretion of pro-inflammatory cytokines such as IL-6, IL-8, IL-1α, and IL-1β [39,40]. Other regulatory factors of the SASP include C/EBPß, GATA4, p38 MAP-kinase, mTOR, JAK/STAT, and Notch pathway [41]. Additionally, several studies have suggested that senomorphic compounds may regulate epigenetic mechanisms that control SASP transcription [42,43]. IL-6 plays a central role in the SASP network [44], IL-8 serves as a chemoattractant cytokine [45], and IL-1 (α and β) initiates signaling cascades that induce IL-6 and IL-8 expression [46]. Hence, suppressing SASP cytokines offers a strategic approach to mitigating the harmful effects of senescence. In this study, compound **3** isolated from the root of *L. formosana* significantly reduced SASP markers (IL-6, IL-8, IL-1α, and IL-1β). These results demonstrate that our compound exerts SASP marker-reducing effects at much lower concentrations than previously reported compounds. Lim et al. reported that avenanthramide C, a compound extracted from oats, suppressed IL-6 and IL-8 expression in senescent human dermal fibroblasts (HDFs) at 120 µM [47]. Other well-studied senomorphic candidates, such as fisetin and quercetin, typically require concentrations above 20 µM to achieve comparable effects [48,49]. Collectively, these benchmarks highlight the superior potency of compound **3** and support its potential as a lead scaffold for the development of senescence-modulating therapeutics.

To further confirm the senescence phenotype, SA-β-gal staining was performed. This histochemical assay uses the chromogenic substrate X-gal, detectable at pH 6.0, which yields an insoluble blue compound when cleaved by β-galactosidase [50]. The results demonstrated that compound **3** exhibits the most promising SASP-inhibitory activity among the isolated compounds. According to our data, compound **3** exhibits superior SA-β-gal inhibition at relatively lower concentrations compared to previously reported compounds or extracts. Previous studies showed that ginsenosides from heat-processed ginseng at 120 °C, when administered to endothelial progenitor cells at 200 μg/mL, reduced SA-β-gal positivity from 93.8% to 62.5% [36]. Extracts from *Silybum marianum* flowers demonstrated senotherapeutic-like effects, significantly decreasing the proportion of SA-β-gal-positive senescent HDFs by 33.2% at 200 μg/mL [37]. Kaempferol-type compounds from *Nephelium lappaceum* were also reported to reduce the proportion of SA-β-gal-positive cells by approximately 50% [38]. Although compound **3** was not evaluated for activity using the same cell system as the previously reported compounds, these results support the potential of compound **3** as a novel senomorphic agent for the treatment of aging-associated diseases by targeting p16^INK4A^ expression, SASP modulation, and morphological features of cellular senescence.

This study has several limitations that should be acknowledged. Our findings were primarily obtained from continuous cell lines, including A549 lung carcinoma cells and IMR90 fibroblasts, which may not fully recapitulate the complexity of primary human tissues or patient-derived senescent cells. Although these models are well established for senescence research, the translational relevance of our results remains to be validated in more physiologically relevant systems. While we demonstrated that compound **3** effectively reduced p16^INK4A^ promoter activity, SASP cytokine expression, and SA-β-gal positivity, its precise molecular targets and upstream regulatory pathways remain undefined (Supplementary Figure S21). Further studies using primary cells, patient-derived models, and in vivo systems will therefore be necessary to confirm the senomorphic activity of compound **3** and to elucidate its mechanism of action. These investigations will not only validate the therapeutic potential of compound **3** but also provide broader insights into the role of monoterpenoids in modulating cellular senescence. Future work should further address the heterogeneity of senescence by testing compound **3** across diverse senescence inducers to better clarify its global molecular effects.

## 5. Conclusions

In this study, by using FBMN powered by the GNPS database, the chemical profile of *L. formosana* was established, revealing the presence of flavonoid glycosides, amides, and triterpenoids. Eight meroterpenoids were further identified by integrating SNAP-MS. Two undescribed compounds (**1** and **2**) were isolated, and their structures were characterized by NMR and other spectroscopic techniques. Evaluation of these compounds demonstrated their ability to inhibit senescence markers, including p16^INK4A^, SASP, and SA-β-gal staining, with compound **3** emerging as the most potent inhibitor. To our knowledge, this is the first report to identify a cinnamic acid-conjugated monoterpennoid from *L. formosana* that targets aging-related biomarkers. These findings highlight the potential as promising therapeutic candidates for aging-associated diseases through the modulation of cellular senescence.

## Figures and Tables

**Table 1:** ^1^H- and ^13^C-NMR spectroscopic data of compounds **1** and **2** in CDCl_3_^a^.

Position	1 *^b^*		2	
	*δ*_H_ (*J* in Hz)	*δ*_C_	*δ*_H_ (*J* in Hz)	*δ*_C_
1	7.37 (m)	129.0	7.37 (m)	129.0
2	7.38 (m)	130.3	7.38 (m)	130.4
3	7.37 (m)	129.0	7.37 (m)	129.0
4	7.52 (m)	128.2	7.53 (m)	128.2
5		134.6		134.5
6	7.52 (m)	128.2	7.53 (m)	128.2
7	7.68 (d, 16.0)	144.6	7.70, (d, 16.0)	144.9
8	6.45 (d, 16.0)	119.0	6.47, (d, 16.0)	118.2
9		166.7		167.0
12	5.70 (d, 8.0)	68.8	4.60 (2H, s)	70.3
13		150.5		129.3
14	2.56 (t, 5.45)	50.9	7.38 (m)	130.7
15		40.7	2.26 (2H, br s)	26.5
16	2.02 (m)	39.7	2.28 (2H, br s)	30.9
17	1.88 (m), 2.45 (m)	33.5		145.8
18	4.93 (br s), 5.11 (br s)	114.4	6.38, (dd, 17.6, 10.8)	138.9
19	1.58 (m), 1.69 (m)	28.1	5.07 (d, 10.8), 5.24 (d, 17.6)	116.1
20	0.73 (3H, s)	22.1	1.71 (3H, s)	14.2
21	1.31 (3H, s)	26.0	5.02 (d, 12.3)	113.3

^a^ Assignments were based on COSY, HSQC, and HMBC experiments. ^b 1^H and ^13^C NMR spectra were acquired at 400 and 100 MHz, respectively.

## Data Availability

The data that support the findings of this study are available from the corresponding author upon reasonable request.

## Supplementary Materials

The following supporting information can be downloaded at https://www.mdpi.com/article/10.3390/nu17213321/s1. Figure S1: The feature based molecular networking of the total extract from the root of *L. formosana*; Figure S2: IR(KBr) spectrum of compound **1**; Figure S3: UV spectrum of compound **1**; Figure S4: ECD spectrum of compound **1**; Figure S5: ^1^H NMR spectrum of compound **1**; Figure S6: ^13^C NMR spectrum of compound **1**; Figure S7: eHSQC spectrum of compound **1**; Figure S8: HMBC spectrum of compound **1**; Figure S9: COSY spectrum of compound **1**; Figure S10: NOESY spectrum of compound **1**; Figure S11: IR(KBr) spectrum of compound **2**; Figure S12: UV spectrum of compound **2**; Figure S13: ^1^H NMR spectrum of compound **2**; Figure S14: ^13^C NMR spectrum of compound **2**; Figure S15: eHSQC spectrum of compound **2**; Figure S16: HMBC spectrum of compound **2**; Figure S17: COSY spectrum of compound **2**; Figure S18: High-confidence subnetwork (component index 5) and representative compounds predicted by SNAP-MS; Figure S19: Conformers of compound **1**; Figure S20: Bioactivity-guided isolation workflow targeting cellular senescence modulators; Figure S21: Cell viability of bleomycin-treated IMR90 fibroblasts in response to compound **3**; Figure S22: Proposed mechanism of compound **3** action in the p16INK4A pathway; Table S1: Energies and atomic Cartesian coordinates of geometry-optimized conformers of compound **1** at B3LYP/6-31g(d) in methanol. Table S2: Calculated excited state transition wavelengths, oscillator strengths and rotatory strengths for geometry-optimized conformers of compound **1** at B3LYP/6-31g(d) in methanol.

## Abbreviations

The following abbreviations are used in this manuscript:

CCFs	Cytoplasmic chromatin fragments
ECD	Electronic circular dichroism
GNPS	Global natural products social molecular networking
HMBC	Heteronuclear multi-bond correlation
HPLC	High-performance liquid chromatography
HRESIMS	High-resolution electrospray ionization mass spectrometry
HSQC	Heteronuclear single quantum coherence
LC-MS	Liquid chromatography–mass spectrometry
NMR	Nuclear magnetic resonance
SASP	Senescence-associated secretory phenotype
STAT3	Signal transducer and activator of transcription 3
