# Supplementary material for: Monoterpenoids from the Roots of Liquidambar formosana (Formosan Sweet Gum) Exhibit Senomorphic Activity Against Cellular Senescence
Source: Nutrients. 2025 Oct 22;17(21):3321. doi: 10.3390/nu17213321 (PMC12608459; doi:10.3390/nu17213321)
Supplement: Supplementary file 1 [file nutrients-17-03321-s001.zip › nutrients-3867492-supplementary.pdf]

Supplementary Material

Monoterpenoids from the Roots of *Liquidambar formosana* (Formosan Sweet Gum) Exhibit Senomorphic Activity against Cellular Senescence

Minh Thi Tuyet Le [†], Quang Huy Vu [†], Van-Hieu Mai, Jorge Eduardo Ponce-Zea, Seri Choi, Jin-Pyo An, and Won-Keun Oh [*]

Research Institute of Pharmaceutical Sciences, College of Pharmacy, Seoul National University, Seoul 151-742, Republic of Korea; lethituyetminh19289@gmail.com (M.T.T.L.); 2023-23730@snu.ac.kr (Q.H.V.) maihieu@snu.ac.kr (V.H.M); jepz210689@snu.ac.kr (J.E.P-Z); loyalmintt2@naver.com (S.C); ntopjp77@gmail.com (J.P.A); wkoh1@snu.ac.kr (W.K.O)

[*] To whom correspondence should be addressed. Tel & Fax: +82-02-880-7872. E-mail: wkoh1@snu.ac.kr

Table of contents

Figure S1. The feature based molecular networking of the total extract from the root of *L.formosana* 3

Figure S2. IR(KBr) spectrum of compound 1 4

Figure S3. UV spectrum of compound 1 5

Figure S4. ECD spectrum of compound 1 6

Figure S5. ^{1}H NMR spectrum of compound 1 (400 MHz, chloroform-d_3) 7

Figure S6. ^{13}C NMR spectrum of compound 1 (100 MHz, chloroform-d_3) 8

Figure S7. eHSQC spectrum of compound 1 (400 MHz, chloroform-d_3) 9

Figure S8. HMBC spectrum of compound 1 (400 MHz, chloroform-d_3)......10

Figure S9. COSY spectrum of compound 1 (400 MHz, chloroform-d_3) 11

Figure S10. NOESY spectrum of compound 1 (400 MHz, chloroform-d_3)......12

Figure S11. IR(KBr) spectrum of compound 2 13

Figure S12. UV spectrum of compound 2......13

Figure S13. ^{1}H NMR spectrum of compound 2 (400 MHz, chloroform-d_3) 14

Figure S14. ^{13}C NMR spectrum of compound 2 (100 MHz, chloroform-d_3) 15

Figure S15. eHSQC spectrum of compound 2 (400 MHz, chloroform-d_3) 16

Figure S16. HMBC spectrum of compound 2 (400 MHz, chloroform-d_3)......17

Figure S17. COSY spectrum of compound 2 (400 MHz, chloroform-d_3) 18

Figure S18. High-confidence subnetwork (component index 5) and representative compounds predicted by SNAP-MS.......19

Figure S19. Conformers of compound 1 20

Figure S20. Bioactivity-guided isolation workflow targeting cellular senescence modulators 20

Figure S21. Cell viability of bleomycin-treated IMR90 fibroblasts in response to compound 3......21

Figure S22. Proposed mechanism of compound 3 action in the p16^{INK4A} pathway 22

Table S1. Energies and atomic Cartesian coordinates of geometry-optimized conformers of compound 1 at B3LYP/6-31g(d) in methanol......23

Table S2. Calculated excited state transition wavelengths, oscillator strengths and rotatory strengths for geometry-optimized conformers of compound 1 at B3LYP/6-31g(d) in methanol 24

Figure S1. The feature based molecular networking of the total extract from the root of *L.formosana*

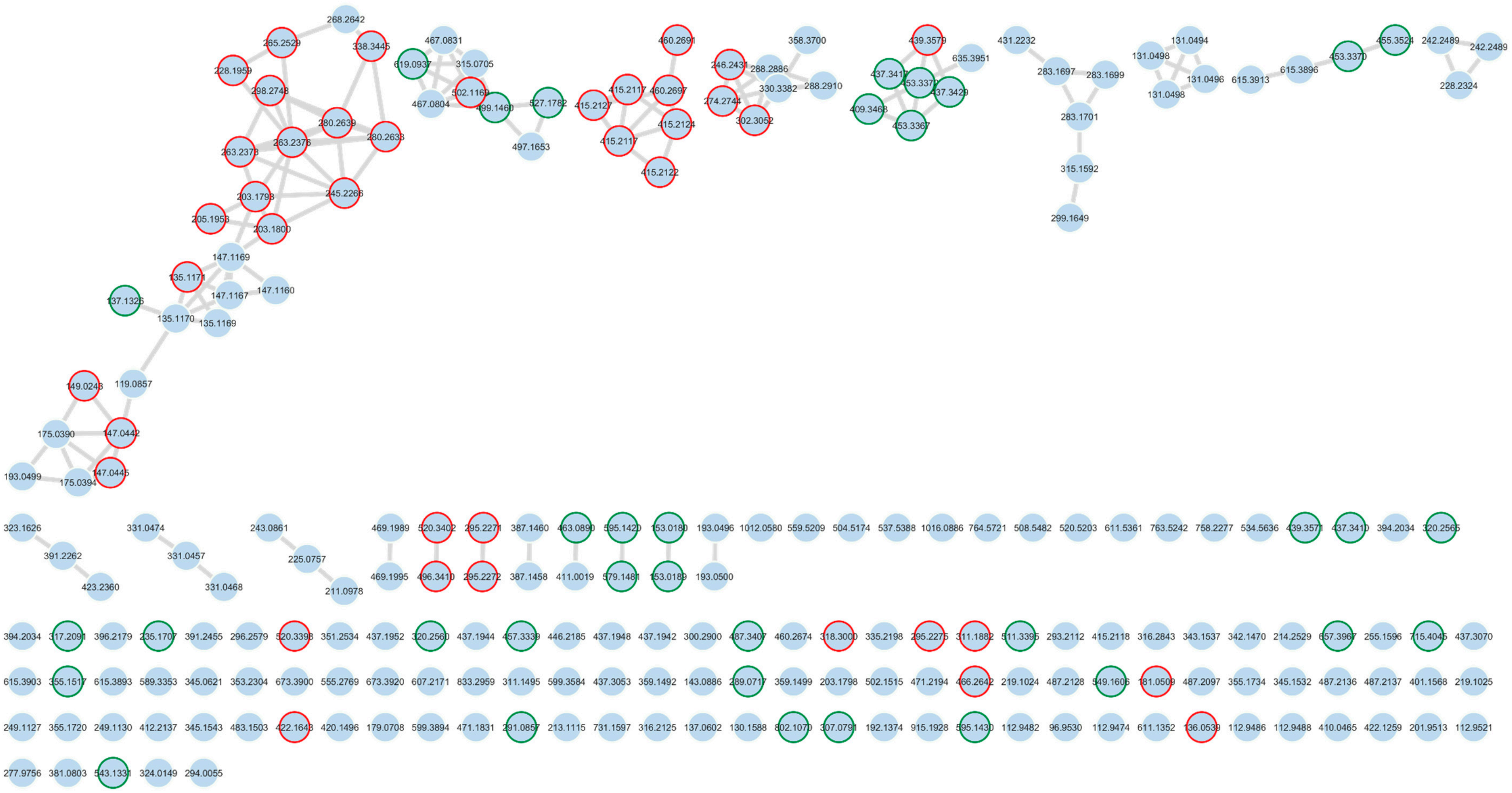

Figure S2. IR(KBr) spectrum of compound **1**

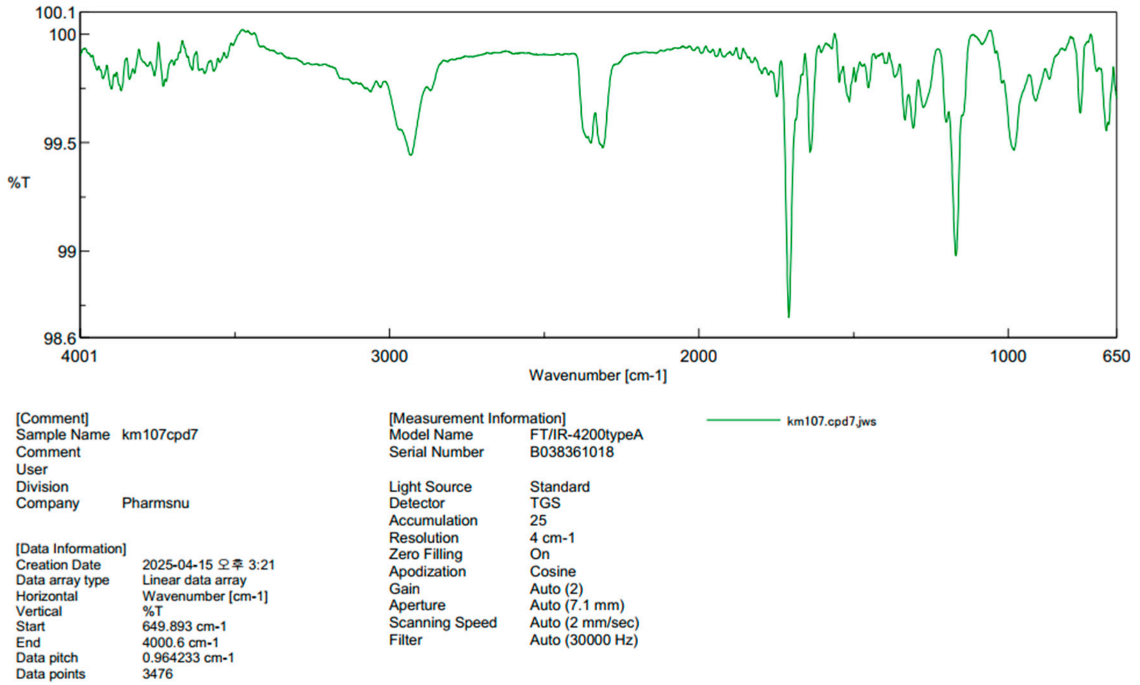

Figure S3. UV spectrum of compound **1**

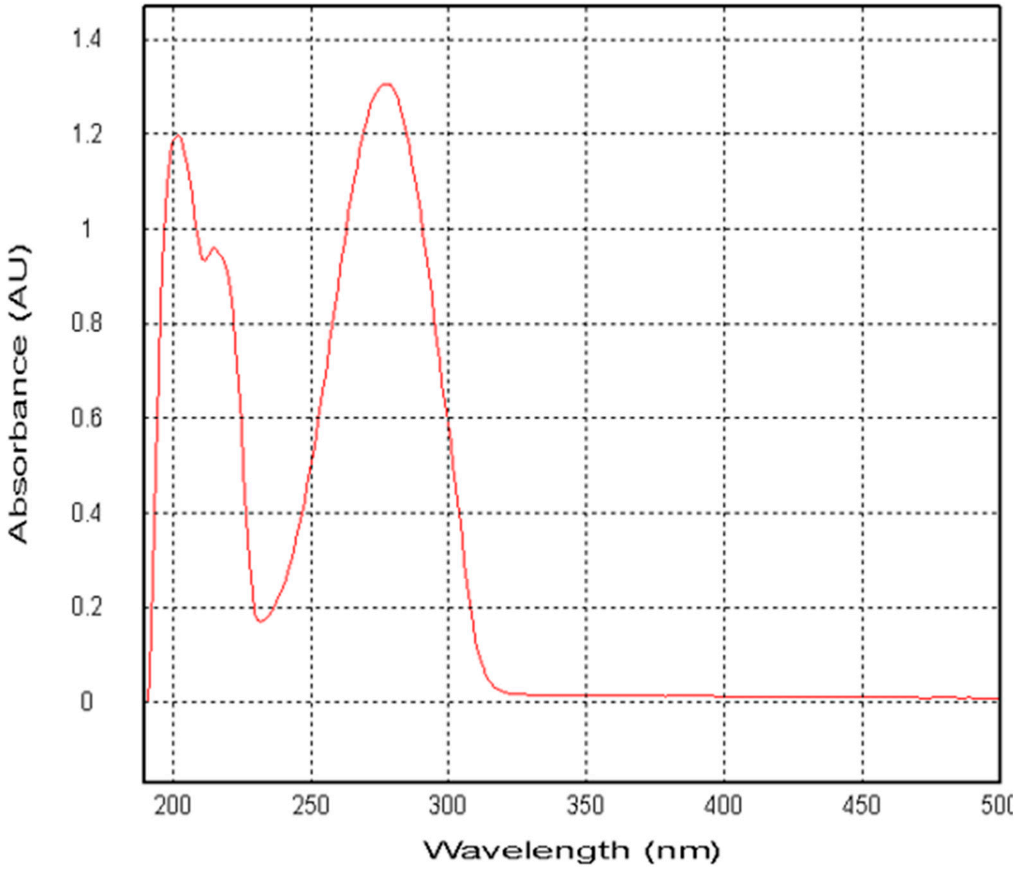

Figure S4. ECD spectrum of compound **1**

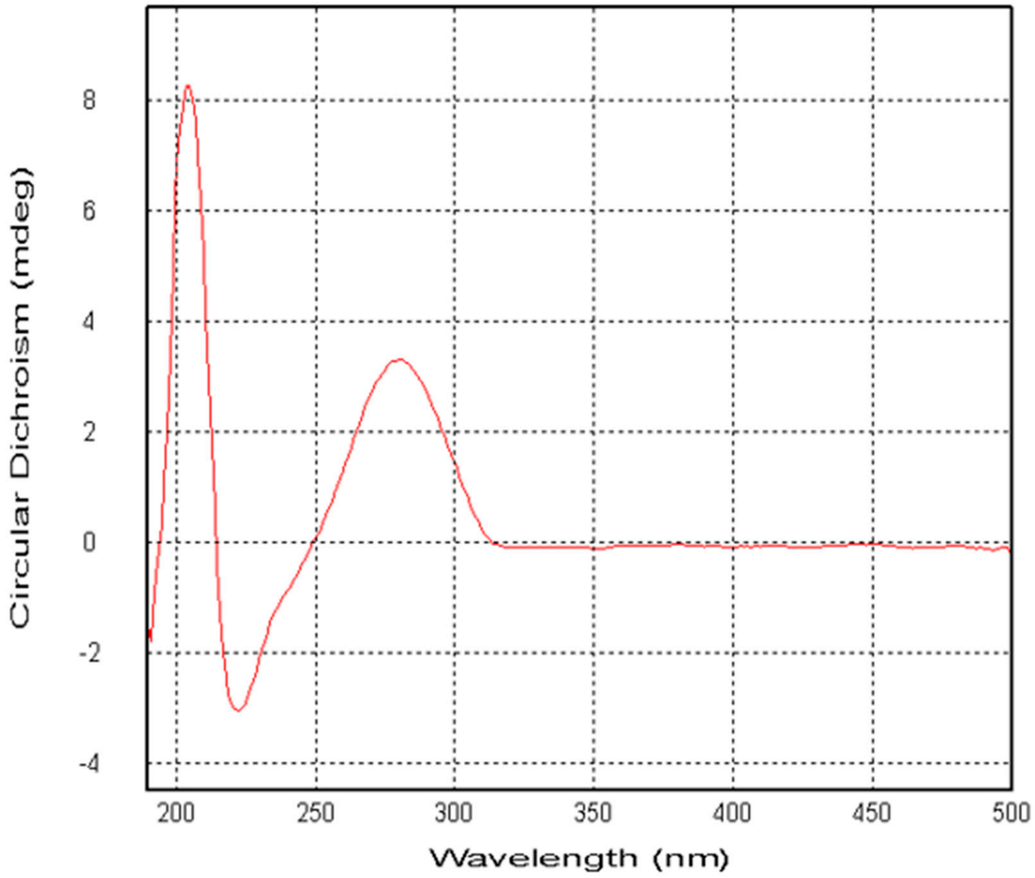

Figure S5. ^{1}H NMR spectrum of compound **1** (400 MHz, chloroform-d_3)

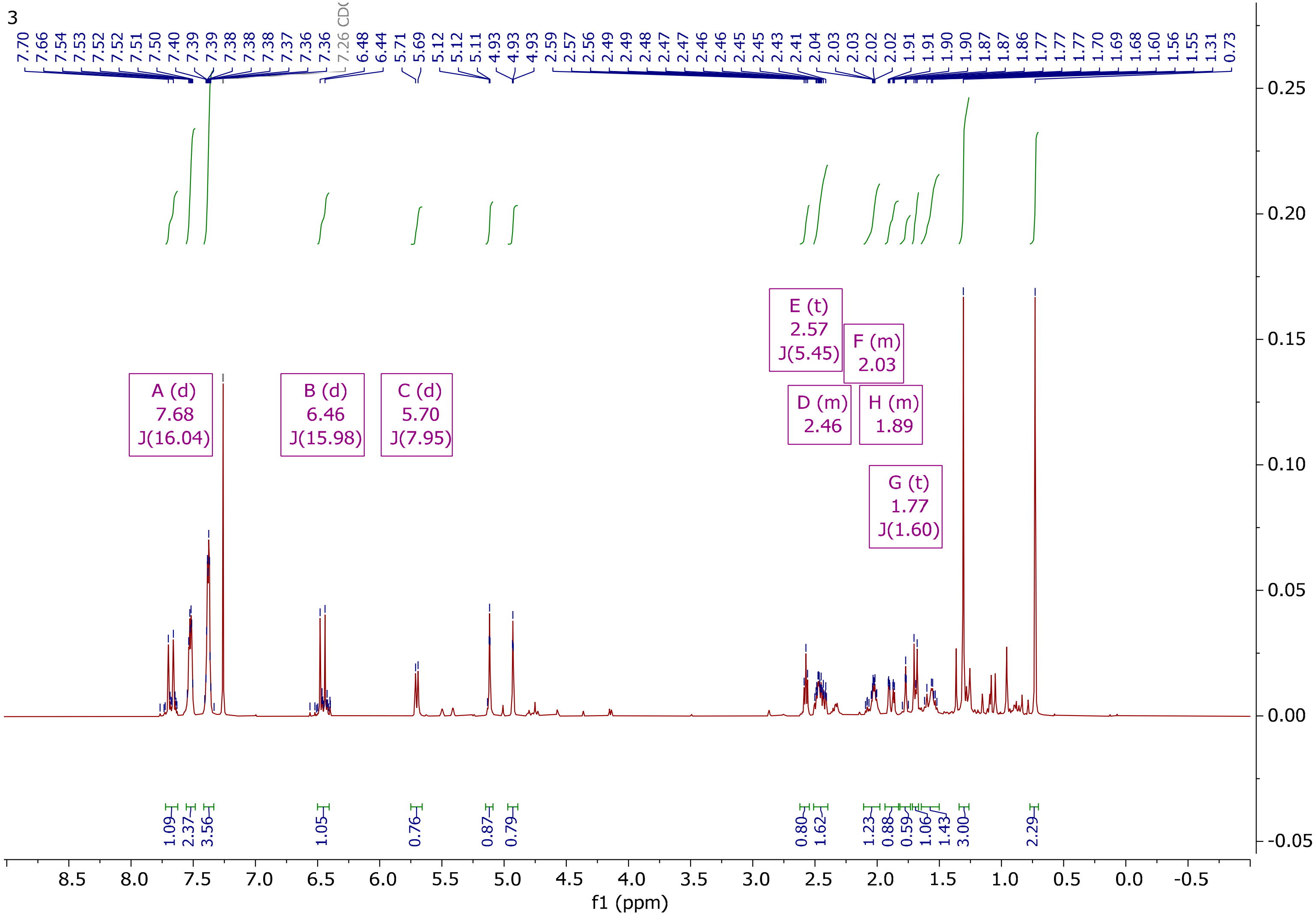

Figure S6. ^{13}C NMR spectrum of compound **1** (100 MHz, chloroform-d_3)

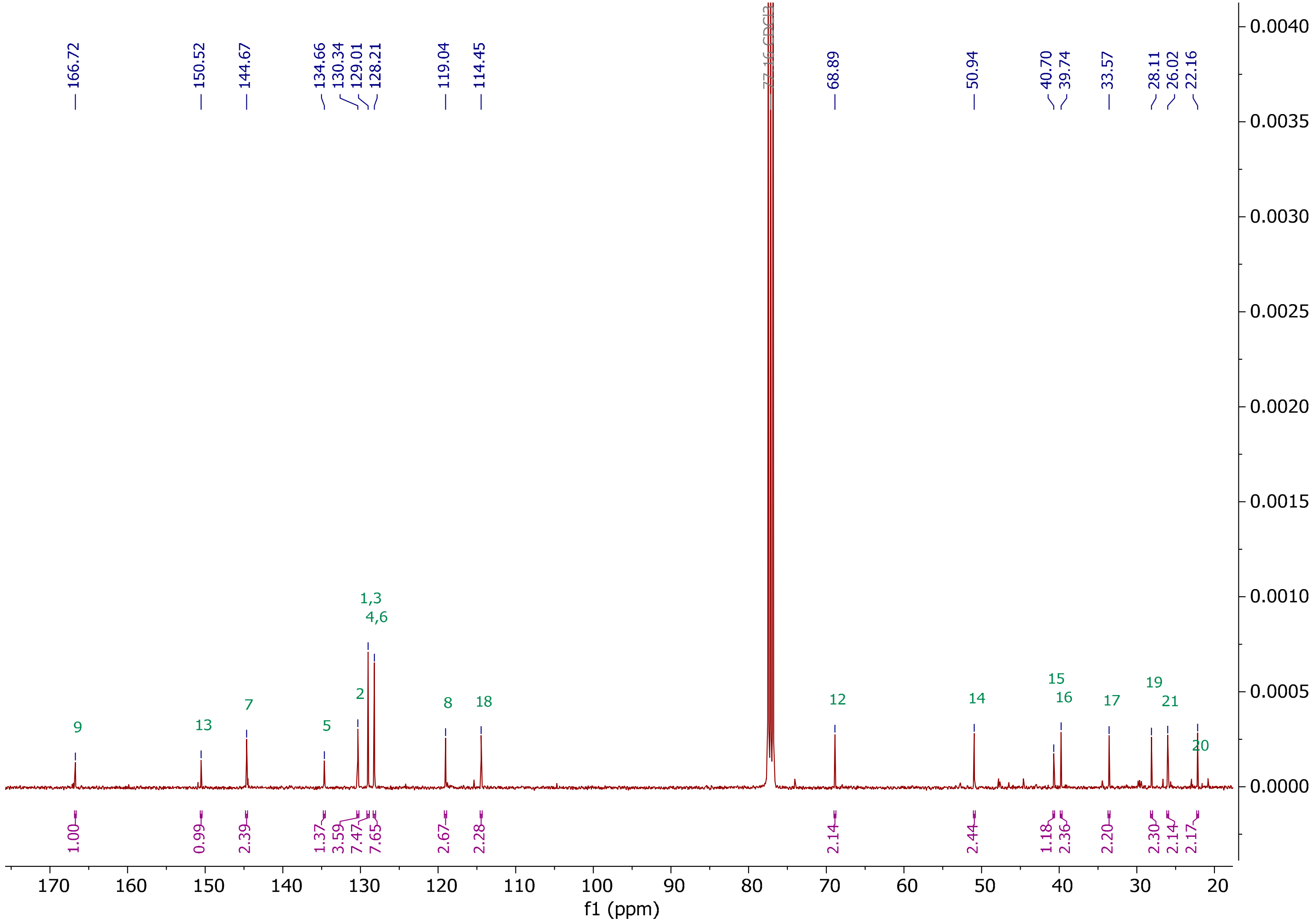

Figure S7. eHSQC spectrum of compound **1** (400 MHz, chloroform-d_3)

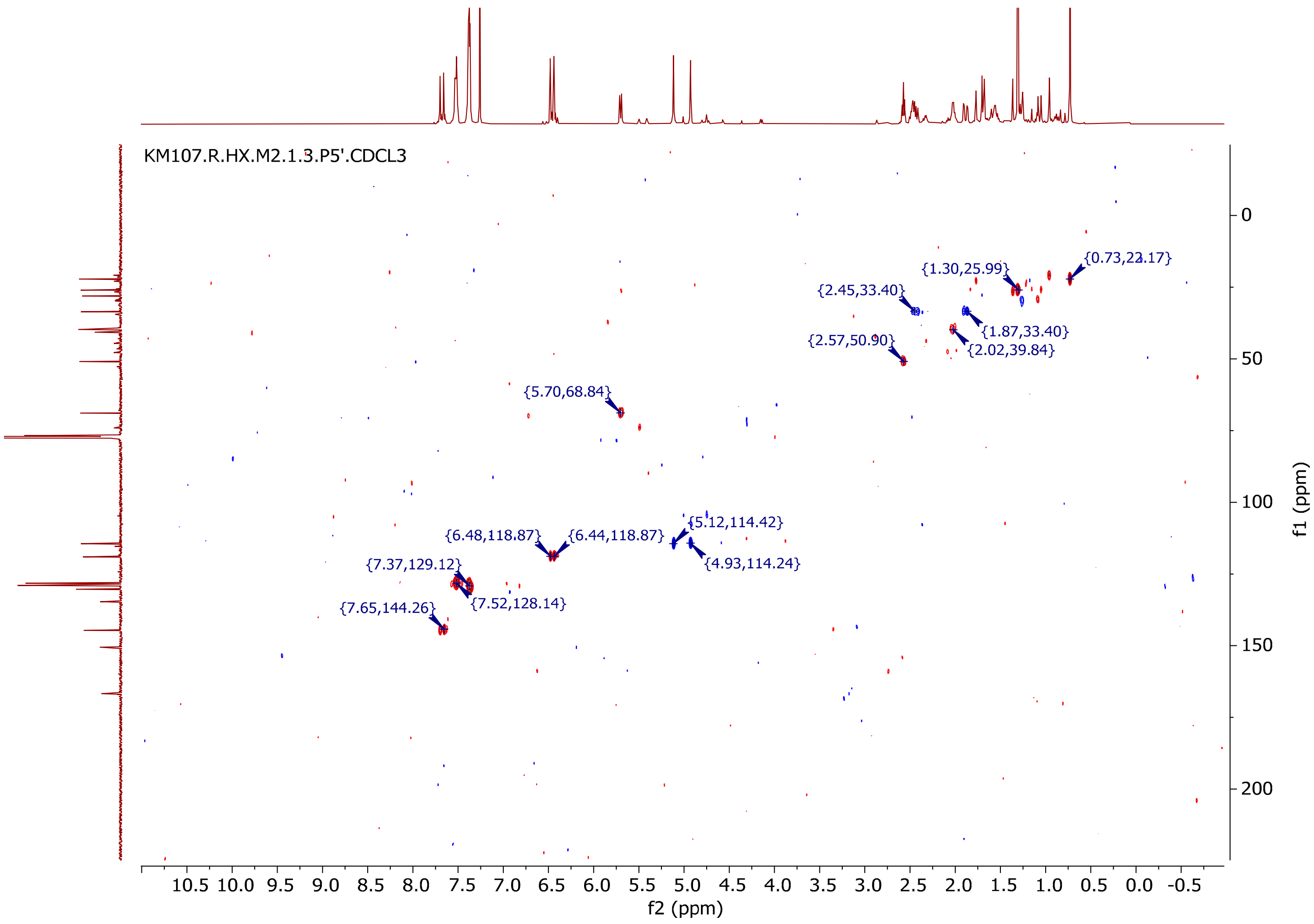

Figure S8. HMBC spectrum of compound **1** (400 MHz, chloroform-d_3)

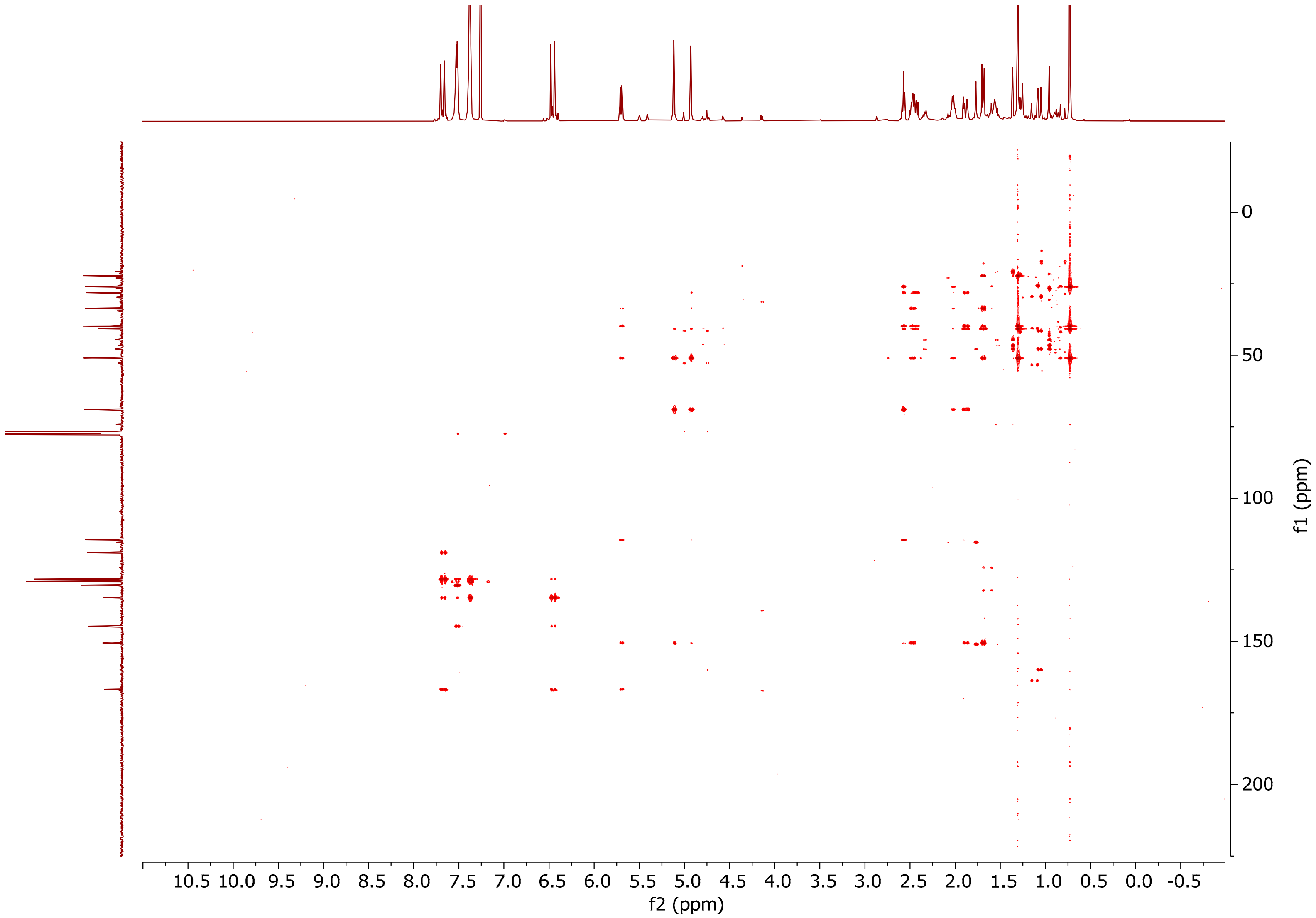

Figure S9. COSY spectrum of compound **1** (400 MHz, chloroform-d_3)

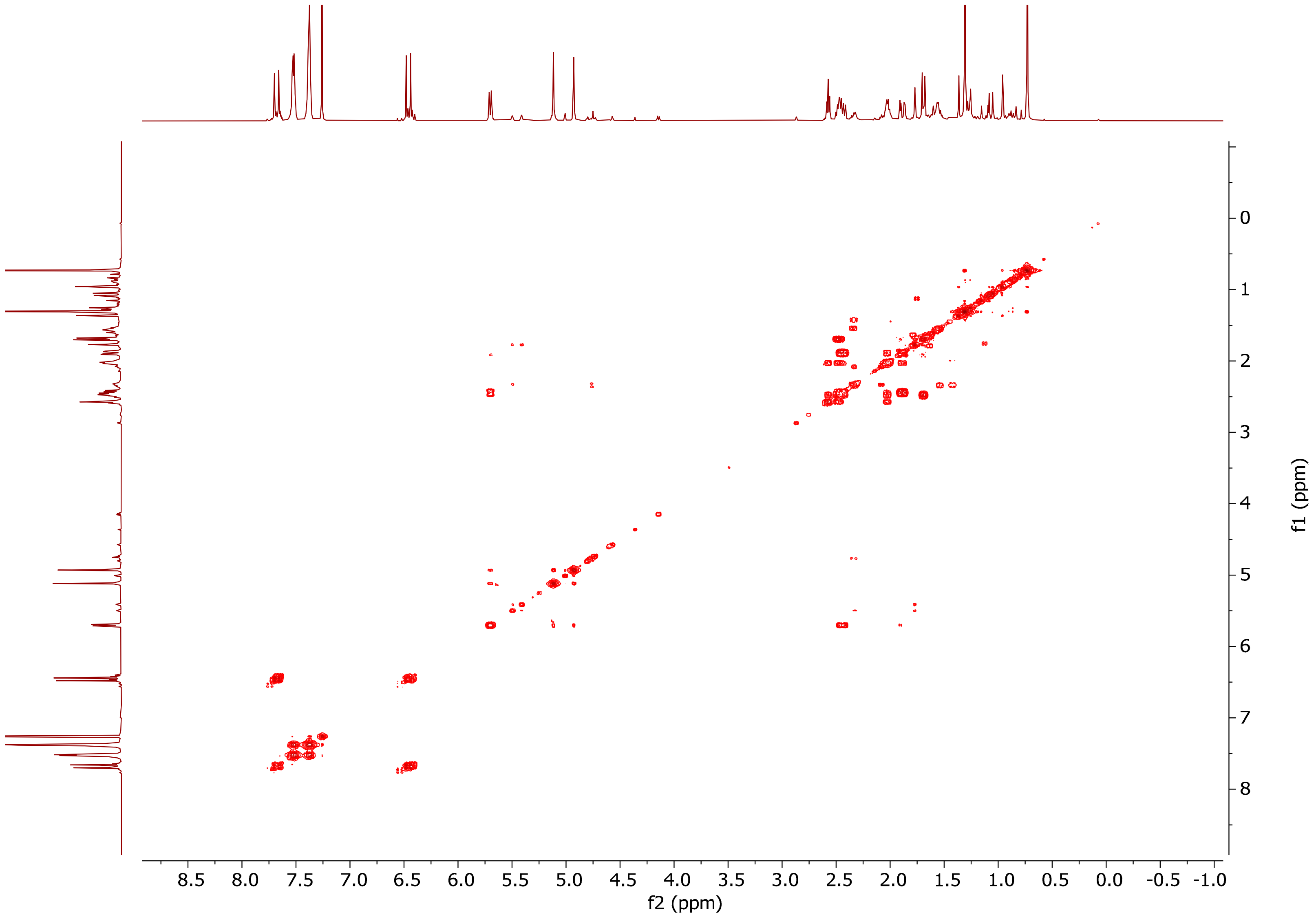

Figure S10. NOESY spectrum of compound **1** (400 MHz, chloroform-d_3)

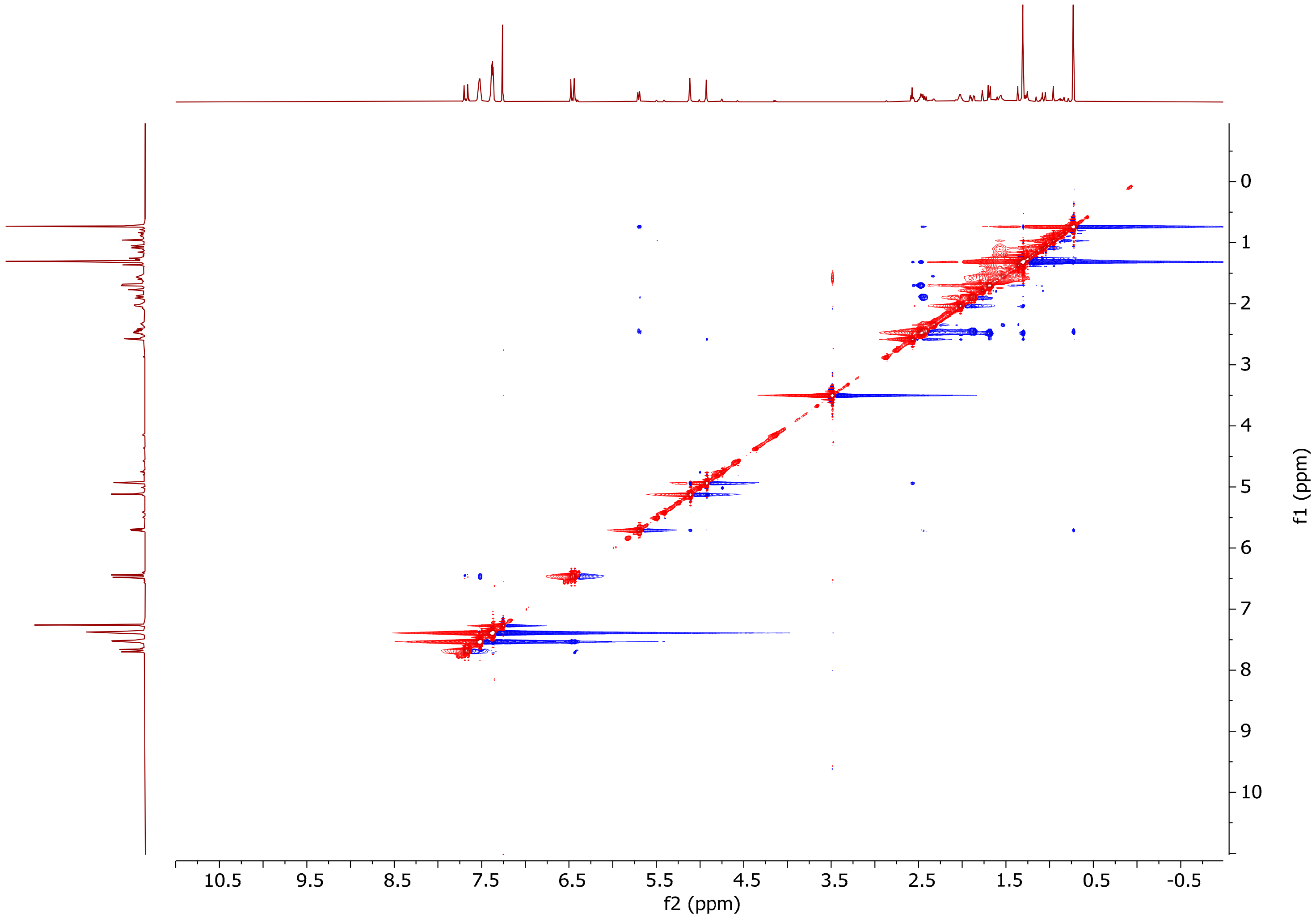

Figure S11. IR(KBr) spectrum of compound **2**

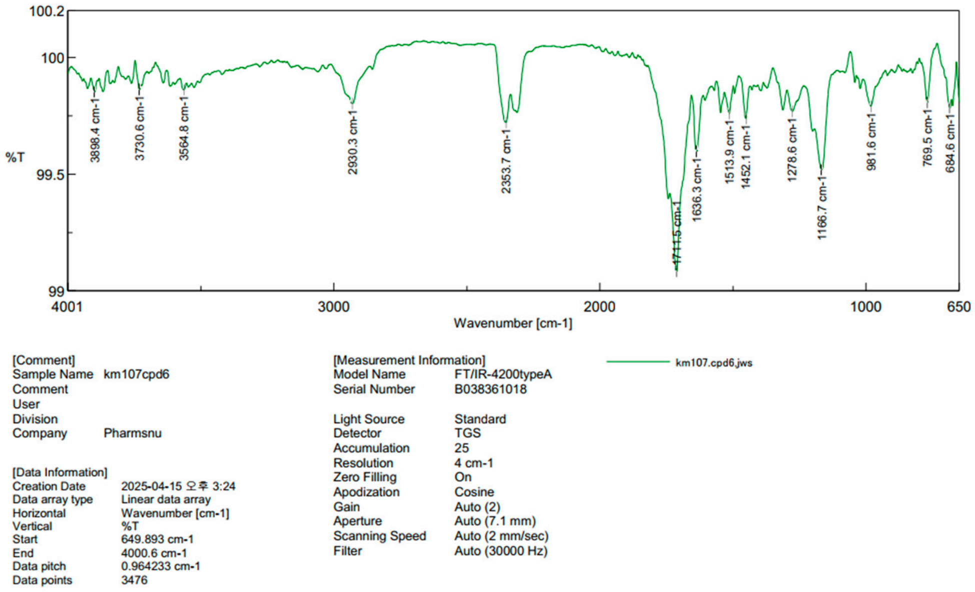

Figure S12. UV spectrum of compound **2**

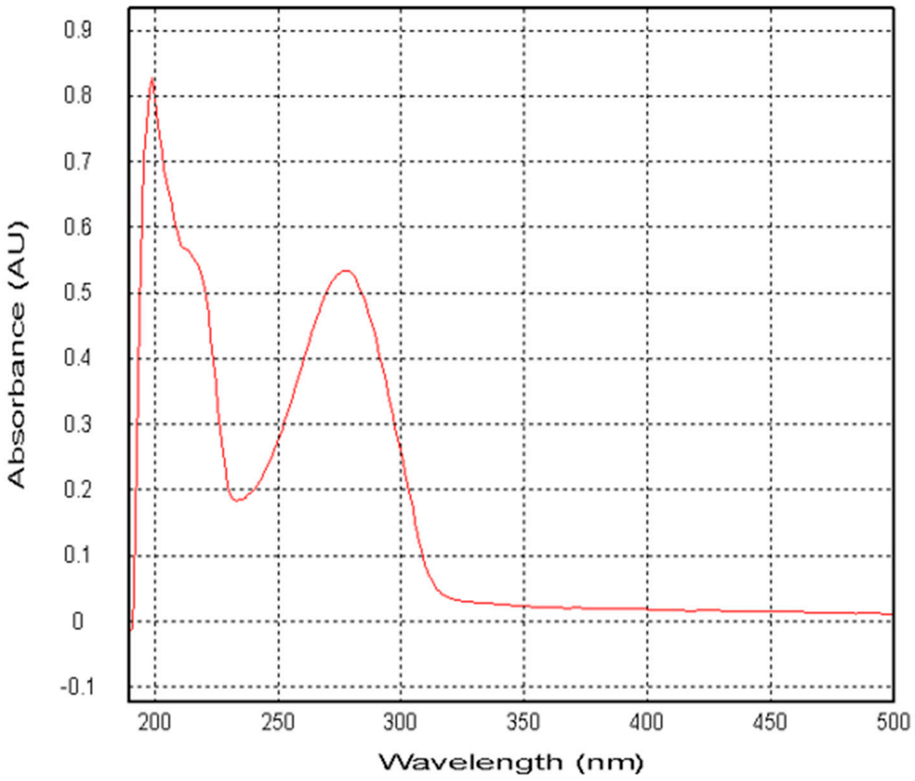

Figure S13. ^{1}H NMR spectrum of compound **2** (400 MHz, chloroform-d_3)

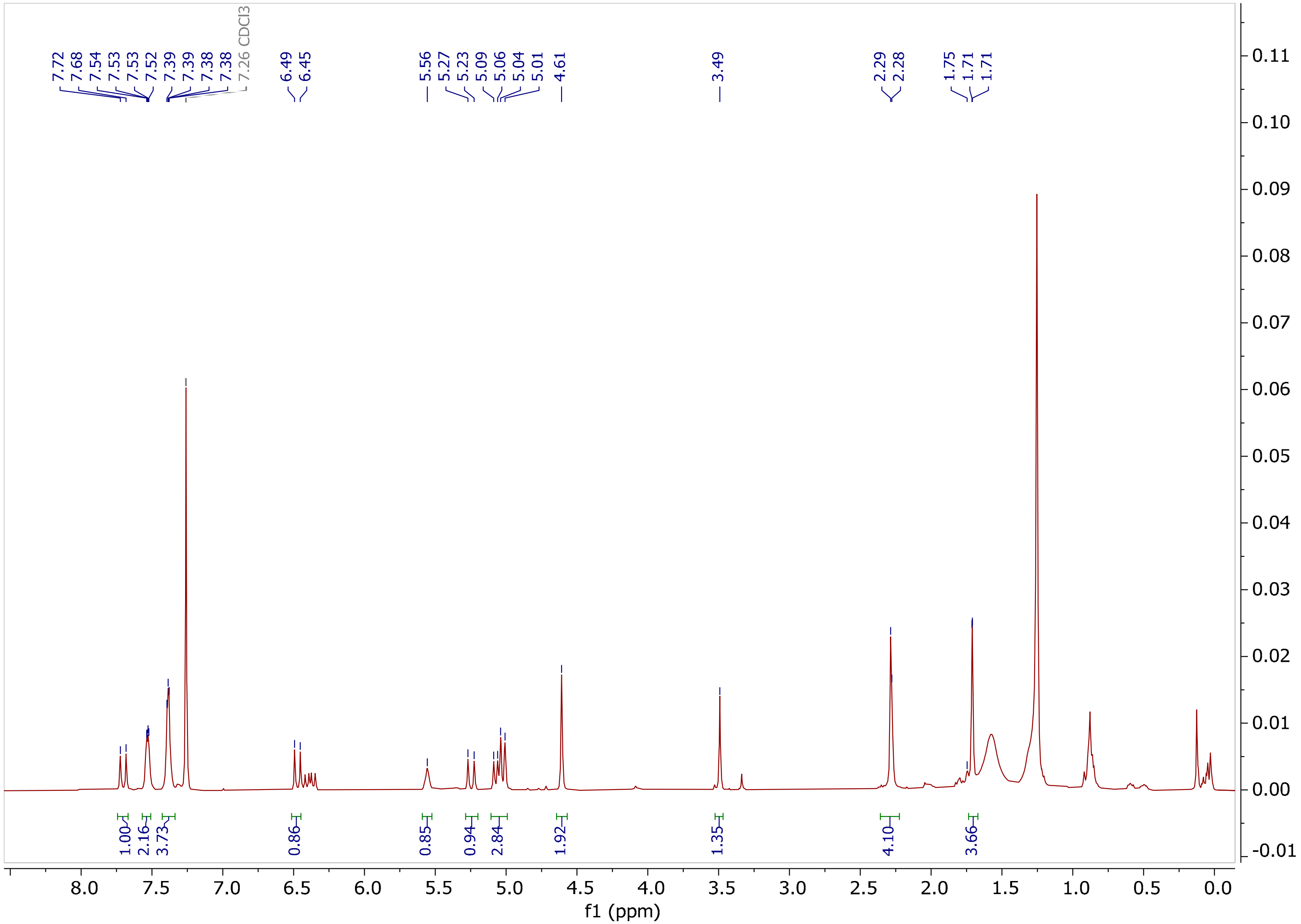

Figure S14. ^{13}C NMR spectrum of compound **2** (100 MHz, chloroform-d_3)

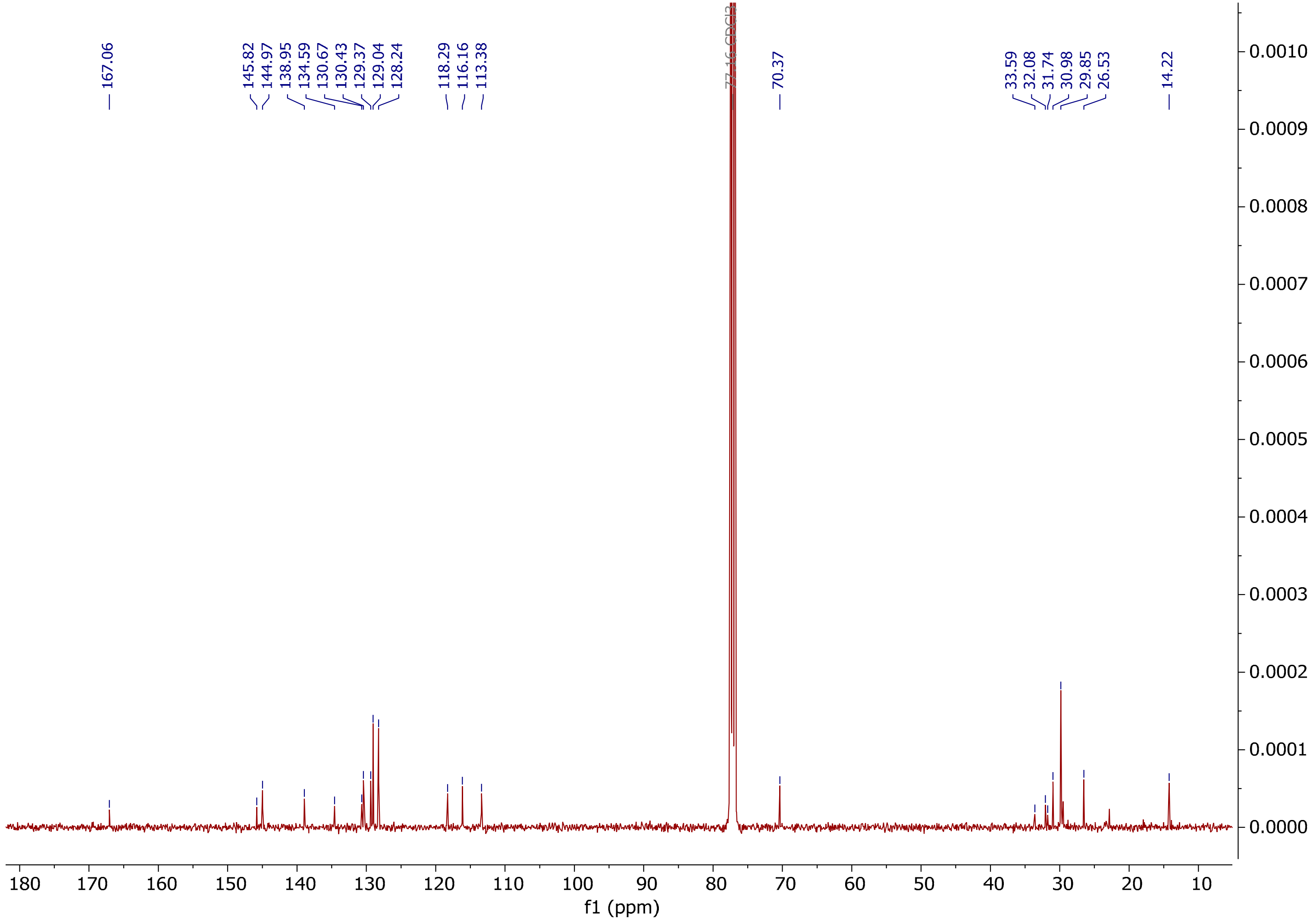

Figure S15. eHSQC spectrum of compound **2** (400 MHz, chloroform-*d*3)

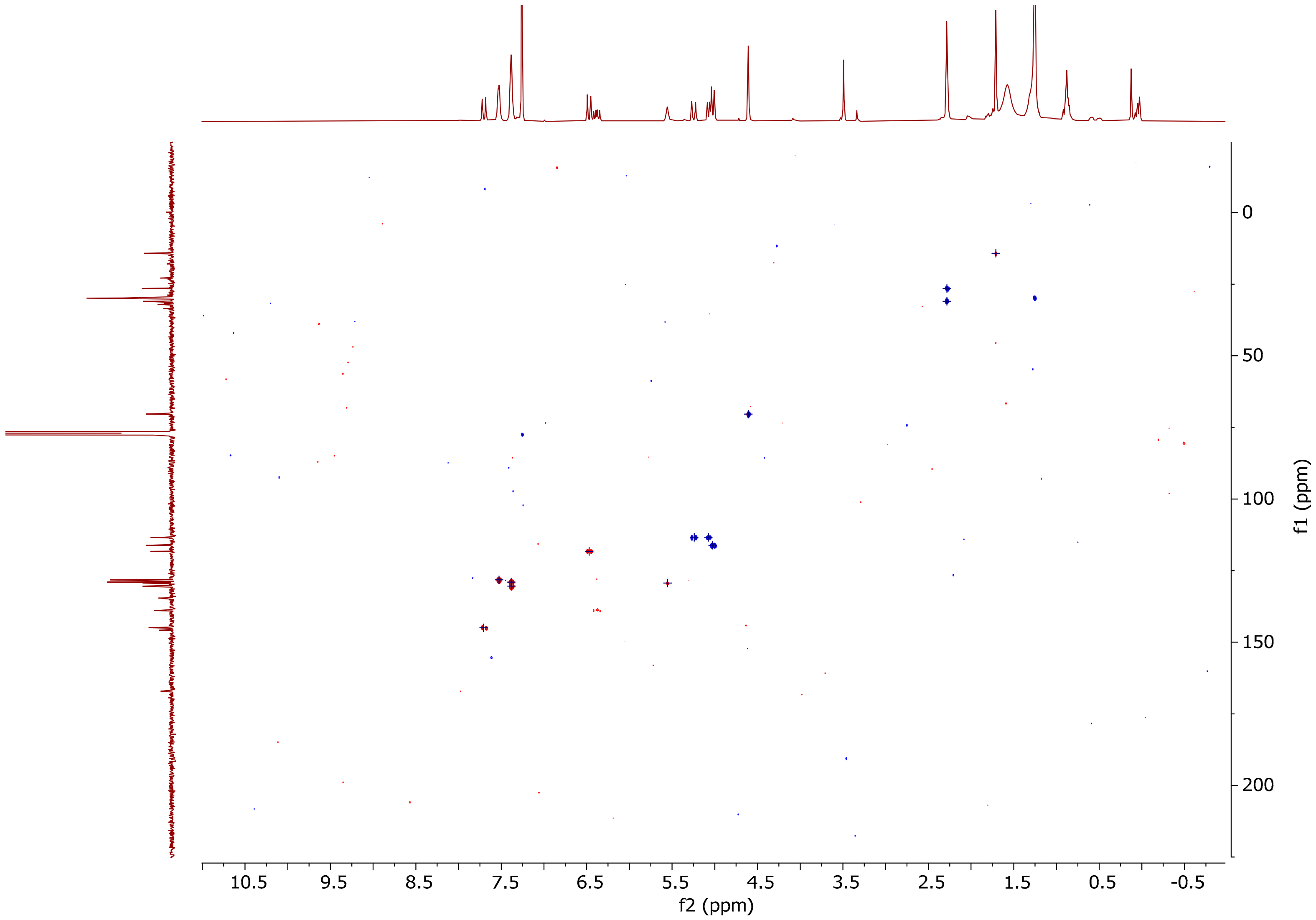

Figure S16. HMBC spectrum of compound **2** (400 MHz, chloroform-d_3)

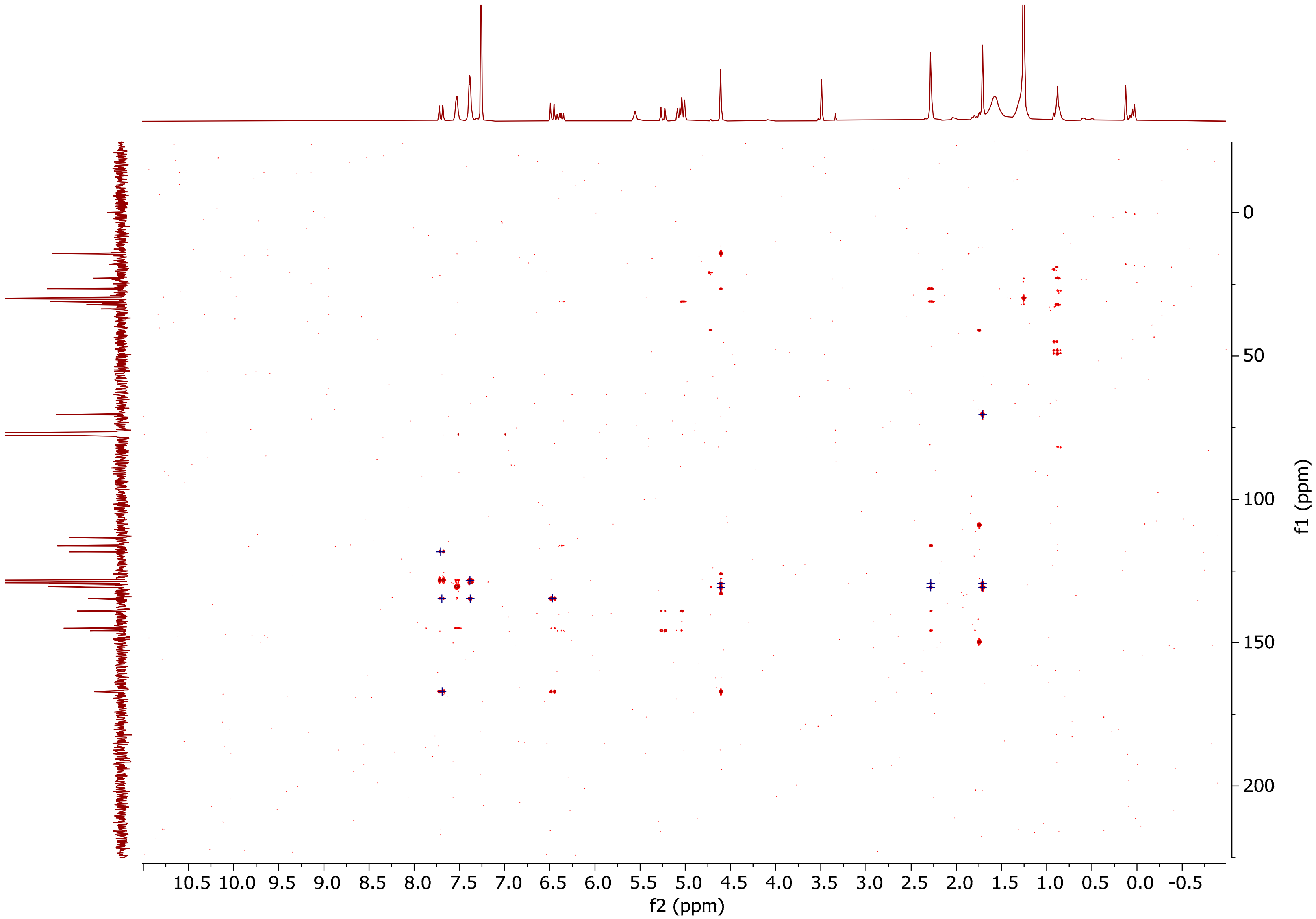

Figure S17. COSY spectrum of compound **2** (400 MHz, chloroform-d_3)

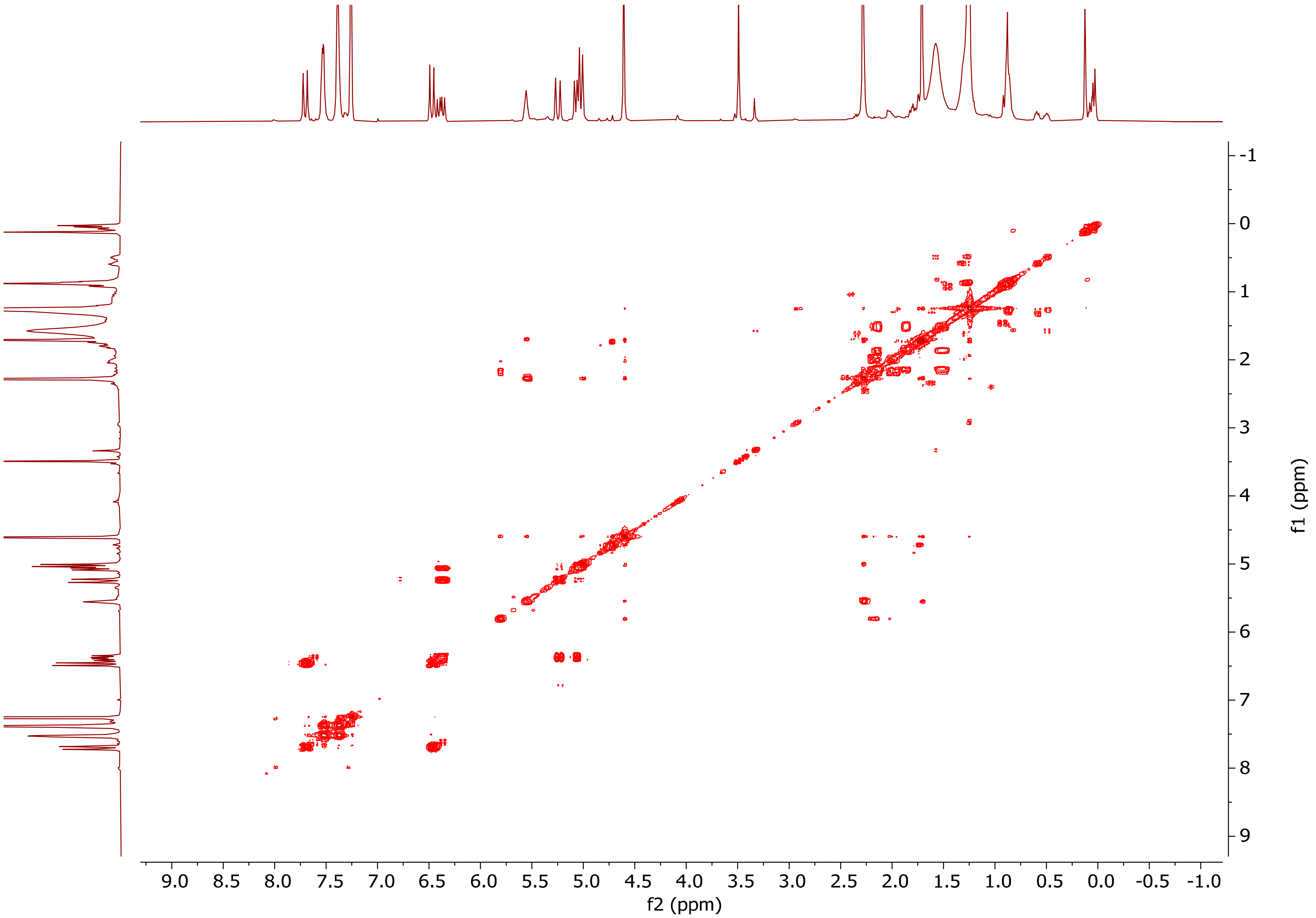

Figure S18. High-confidence subnetwork (component index 5) and representative compounds predicted by SNAP-MS.

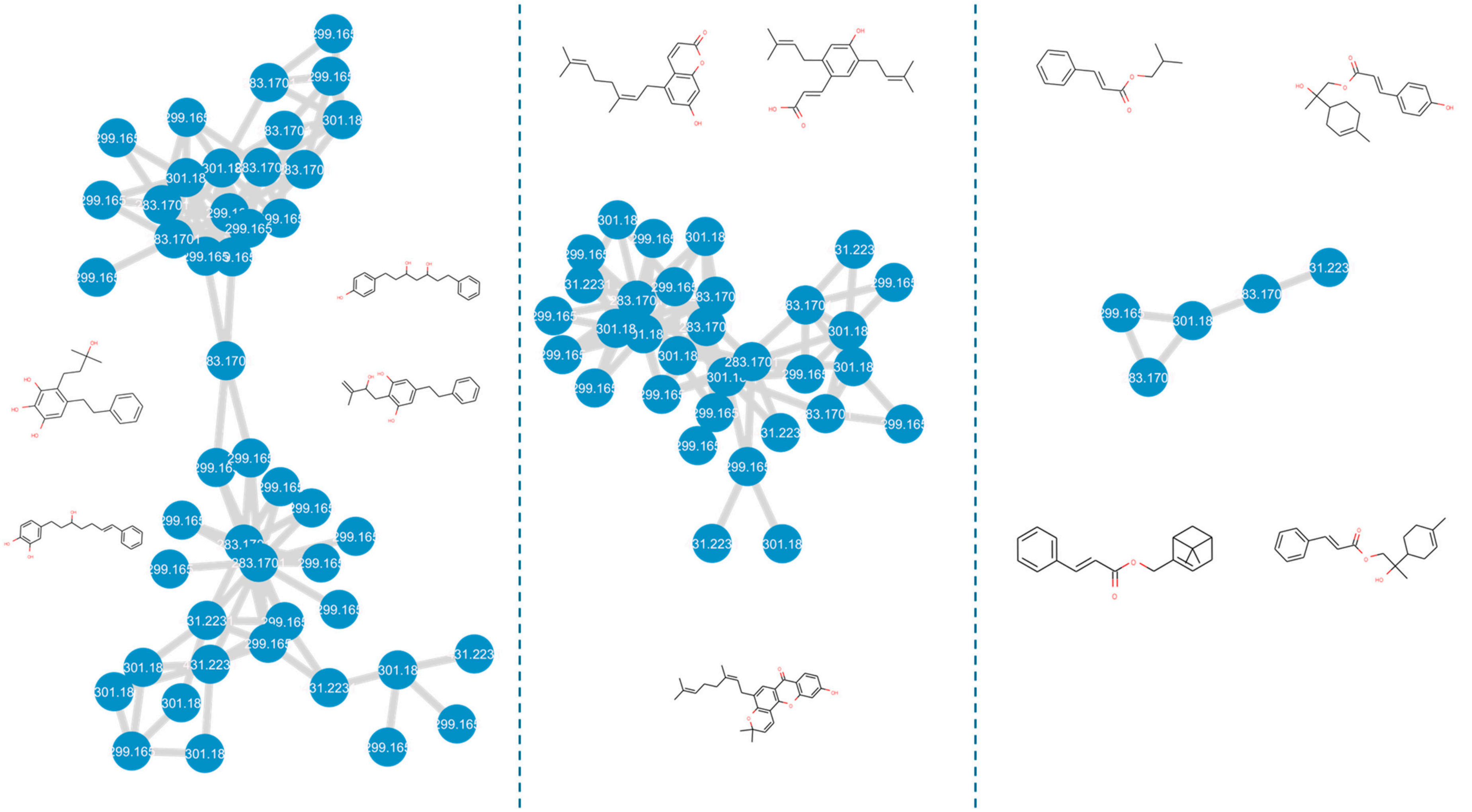

Figure S19. Conformers of compound **1**

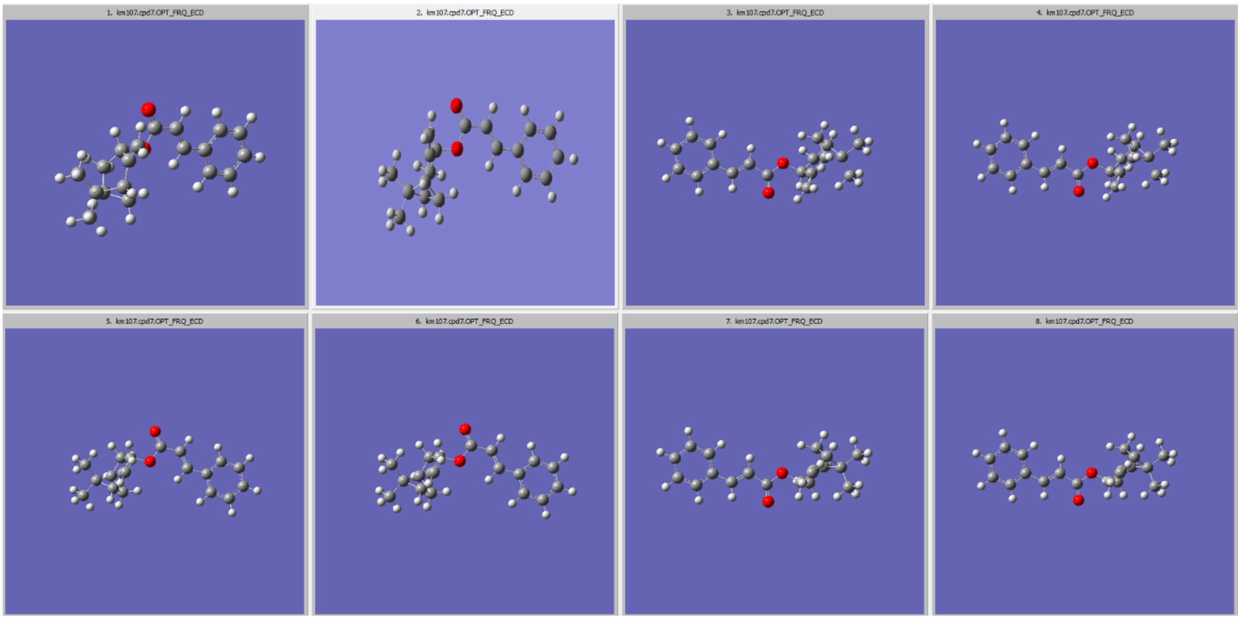

Figure S20. Bioactivity-guided isolation workflow targeting cellular senescence modulators

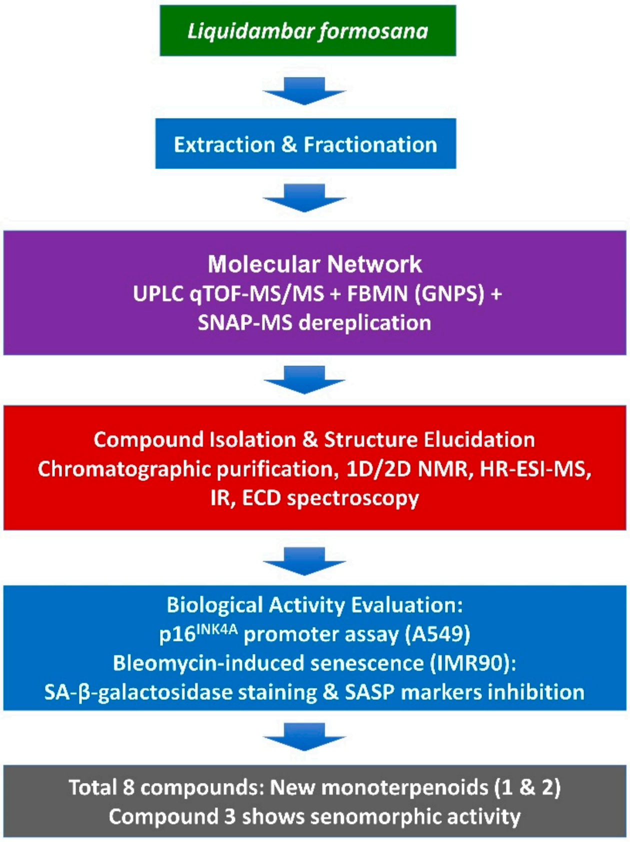

Figure S21. Cell viability of bleomycin-treated IMR90 fibroblasts in response to compound **3**.

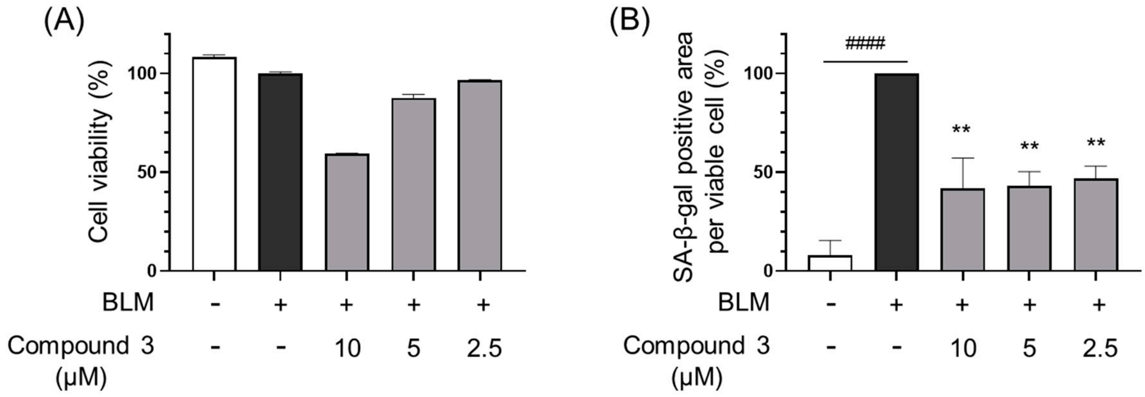

Figure S21. Effect of compound **3** on cell viability and normalized SA-β-gal staining in bleomycin-induced senescent IMR90 fibroblasts. (A) Cell viability was determined by MTT assay after 48 hr of treatment with compound **3** in the presence or absence of bleomycin (BLM, 40 μg/mL). (B) Quantification of SA-β-gal-positive area following 48 hr of treatment with compound **3** after a 2-day BLM pretreatment. SA-β-gal staining results were normalized to cell viability to account for potential cytotoxicity. Data are presented as mean ± SEM (n = 3). Statistical significance was determined by one-way ANOVA followed by Dunn's multiple comparisons test (**$p < 0.01$ vs. 2-day BLM-treated group; ####$p < 0.0001$ vs. control).

Figure S22. Proposed mechanism of compound 3 action in the p16^{INK4A} pathway

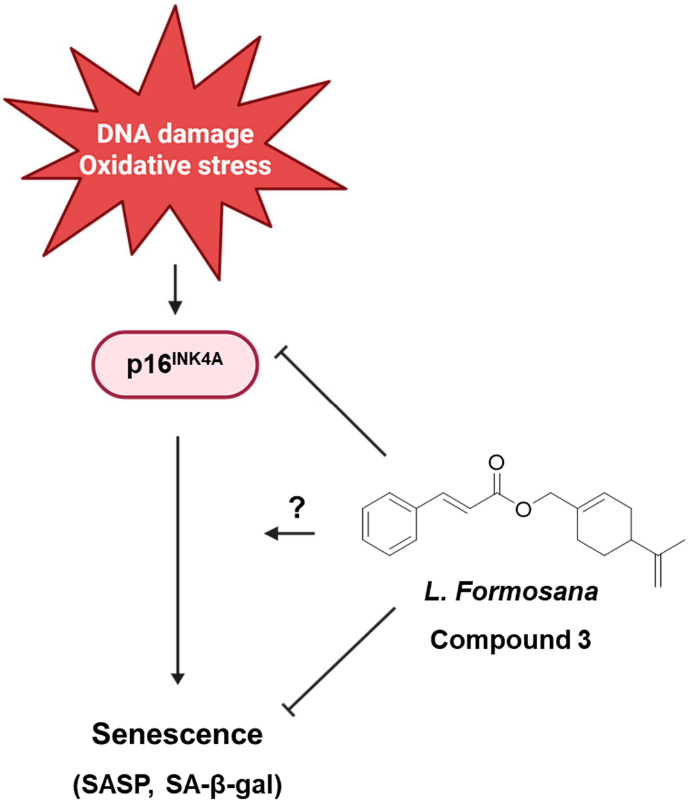

Table S1. Energies and atomic Cartesian coordinates of geometry-optimized conformers of compound **1** at B3LYP/6-31g(d) in methanol

	**Conformer 1**	**Conformer 2**	**Conformer 3**	**Conformer 4**
**Gibbs free energy (hartrees)**	-887.366248	-887.36624	-887.36601	-887.366009
**ΔG (kJ/mol)**		0.021	0.625	0.627
**Population Proportion**	22.23%	22.04%	17.28%	17.26%
	**Conformer 5**	**Conformer 6**	**Conformer 7**	**Conformer 8**
**Gibbs free energy (hartrees)**	-887.365093	-887.365072	-887.364659	-887.364658
**ΔG (kJ/mol)**	3.032	3.088	4.172	4.175
**Population Proportion**	6.54%	6.4%	4.13%	4.13%

Table S2. Calculated excited state transition wavelengths, oscillator strengths and rotatory strengths for geometry-optimized conformers of compound **1** at B3LYP/6-31g(d) in methanol

	**Conformer 1**			**Conformer 2**			**Conformer 3**			**Conformer 4**		
**State**	λ	*f*	*R*	λ	*f*	*R*	λ	*f*	*R*	λ	*f*	*R*
1	294.49	0.1485	20.0643	294.50	0.1481	20.0343	294.52	0.1913	26.878	294.52	0.1914	26.8667
2	286.13	0.7768	-9.235	286.13	0.7773	-9.1594	285.28	0.7427	-28.1597	285.28	0.7427	-28.2008
3	274.96	0.0252	-1.0352	274.96	0.0252	-1.0313	274.77	0.0258	0.1174	274.77	0.0258	0.1168
4	270.90	0.0058	10.2496	270.90	0.0059	10.2954	266.32	0.0014	7.9856	266.32	0.0013	7.9716
5	234.84	0.0158	-9.0461	234.84	0.0158	-9.0629	233.95	0.016	4.0027	233.96	0.016	4.0068
6	225.88	0.0121	1.576	225.89	0.012	1.6056	222.87	0.0189	4.0152	222.88	0.0189	4.0121
7	218.12	0.1157	-14.5647	218.12	0.1156	-14.6504	217.99	0.1188	-8.1321	218.00	0.1188	-8.1226
8	217.66	0.0001	-0.1753	217.68	0.0001	-0.0975	217.46	0.0002	-0.3445	217.46	0.0002	-0.3418
9	209.23	0.03	-1.4945	209.24	0.03	-1.514	208.45	0.0299	1.2409	208.45	0.0299	1.2436
10	203.96	0.0222	0.9532	203.95	0.0225	0.9301	203.85	0.0481	8.8599	203.85	0.0483	8.872
11	203.50	0.001	1.2824	203.51	0.001	1.2868	203.31	0.0091	1.3723	203.30	0.0089	1.3584
12	203.01	0.0405	3.2852	203.00	0.0402	3.3223	202.93	0.0001	0.4662	202.93	0.0001	0.4699
13	194.36	0.1753	32.8063	194.36	0.1753	32.6987	195.12	0.1606	35.7682	195.12	0.1604	35.7577
14	193.46	0.0115	-6.206	193.46	0.0114	-6.1802	194.39	0.0378	-21.1312	194.39	0.0379	-21.1521
15	191.83	0.0	0.1877	191.82	0.0	0.2021	191.74	0.0	0.1025	191.74	0.0	0.0994
16	189.92	0.1393	-9.2507	189.92	0.1391	-9.0853	187.28	0.2523	38.01	187.27	0.252	38.2548
17	185.21	0.0097	-12.1858	185.21	0.0099	-12.294	186.12	0.0164	-25.566	186.12	0.0168	-25.8076
18	184.80	0.0022	-0.2345	184.80	0.0021	-0.1546	184.61	0.045	-18.653	184.61	0.045	-18.6461
19	183.93	0.0455	-8.7232	183.93	0.0455	-8.8433	184.46	0.0011	-1.4457	184.46	0.0012	-1.4777
20	181.91	0.0345	27.7413	181.91	0.0344	28.0616	181.65	0.0002	0.2674	181.65	0.0002	0.2683

	**Conformer 5**			**Conformer 6**			**Conformer 7**			**Conformer 8**		
**State**	λ	*f*	*R*	λ	*f*	*R*	λ	*f*	*R*	λ	*f*	*R*
1	297.14	0.1223	51.5841	297.12	0.1227	51.6601	296.17	0.1105	42.6701	296.17	0.1106	42.6612
2	285.30	0.7205	-61.1129	285.28	0.7201	-60.9885	286.22	0.7199	-59.1969	286.21	0.72	-59.0054
3	274.89	0.0226	-0.8859	274.89	0.0226	-0.8816	275.10	0.0199	-2.2703	275.10	0.0199	-2.2543
4	268.72	0.0077	25.7727	268.70	0.0076	25.6231	273.56	0.0248	38.8903	273.56	0.0246	38.7582
5	235.39	0.0087	2.9671	235.38	0.0087	2.9638	236.32	0.0088	-4.2571	236.33	0.0088	-4.2509
6	224.16	0.0052	-0.7746	224.15	0.0052	-0.7671	227.31	0.0037	-3.084	227.30	0.0037	-3.0815
7	218.42	0.0004	0.3749	218.42	0.0004	0.3707	218.78	0.0009	-0.1407	218.78	0.0008	-0.1697
8	218.09	0.1047	13.0858	218.09	0.1048	13.0781	218.17	0.1041	7.4497	218.16	0.1041	7.4662
9	206.38	0.0686	11.0184	206.37	0.0686	10.9488	207.61	0.0456	-21.9825	207.61	0.0456	-21.9666
10	205.79	0.0065	3.2723	205.79	0.0065	3.2566	205.52	0.0153	7.4049	205.53	0.0154	7.3962
11	204.90	0.003	0.2988	204.90	0.003	0.3544	204.08	0.0003	-2.2881	204.08	0.0003	-2.2851
12	204.01	0.002	-0.7377	204.01	0.002	-0.7437	203.81	0.0246	10.7543	203.81	0.0245	10.7452
13	195.09	0.1059	4.3981	195.08	0.1086	5.1511	194.42	0.1961	36.5803	194.42	0.1962	36.6423
14	194.71	0.0811	40.0873	194.70	0.0786	39.3499	193.70	0.001	0.958	193.70	0.001	0.9348
15	191.81	0.0	-0.0418	191.80	0.0	-0.0517	192.18	0.0006	1.1726	192.17	0.0006	1.1717
16	187.95	0.1691	50.2502	187.94	0.1707	50.1405	190.46	0.1079	-18.2797	190.45	0.1079	-18.3953
17	187.02	0.1166	-51.804	187.02	0.1147	-51.7873	185.98	0.0019	-3.6718	185.98	0.0019	-3.7516
18	185.41	0.031	-20.4605	185.41	0.0312	-20.4574	185.22	0.0652	-17.0498	185.21	0.0652	-16.9152
19	183.01	0.0022	4.977	183.01	0.0022	4.9585	183.00	0.0034	-6.2969	183.00	0.0034	-6.2324
20	182.43	0.0002	0.1752	182.43	0.0002	0.1621	182.82	0.0012	-4.1283	182.81	0.0012	-4.1283

λ = wavelength (nm), *f* = oscillator strength, *R* = rotatory strength (× 10^{-40} esu cm erg/G).
